# Digital Physiotherapeutic Ankle-Specific Training System for Patients With Chronic Ankle Instability Following Modified Brostrom Surgery: Noninferiority Randomized Controlled Trial at a Tertiary Grade A Trauma Center in China

**DOI:** 10.2196/78307

**Published:** 2025-12-18

**Authors:** Xiang Geng, Yilin Jin, Xu Wang, Xin Zhang, Yiming Lyu, Shengdi Lu, Xin Ma

**Affiliations:** 1 Department of Orthopedic Surgery, Huashan Hospital, Fudan University, No. 12, Middle Wulumuqi Road, Shanghai, 200040, China; 2 School of Nursing, Fudan University, Shanghai, China; 3 Rehabilitation Center, School of Medicine, Tongji University, Shanghai, China; 4 Department of Orthopedics, Medmotion Clinic, Shanghai, China; 5 Department of Orthopedics, Shanghai Sixth People's Hospital Affiliated to Shanghai Jiao Tong University School of Medicine, Shanghai, China

**Keywords:** chronic ankle instability, digital training system, internet-based intervention, cost-effectiveness analysis, randomized controlled trial

## Abstract

**Background:**

Functional rehabilitation is commonly used for patients with chronic ankle instability (CAI). Digital training (DT) systems have become increasingly popular in postoperative rehabilitation; however, their effectiveness for CAI patients after modified Brostrom surgery (MBS) is uncertain. Furthermore, specialized physiotherapy resources for CAI are limited in some regions, highlighting the need for effective digital home-based rehabilitation alternatives.

**Objective:**

This trial aimed to evaluate whether individually tailored physiotherapeutic ankle-specific training (PAST) delivered via a DT system is noninferior to conventional face-to-face physiotherapy in clinical outcomes and to compare their cost-effectiveness in CAI patients following MBS in China.

**Methods:**

We conducted a 2-arm, single-blinded (assessor), noninferiority randomized controlled trial at a tertiary hospital in Shanghai, China, from January 2022 to January 2024. A total of 84 postsurgery CAI patients were randomly assigned to a DT group (n=42), which received a 12-week individualized PAST program via a digital system, or a physiotherapy group (n=42), which received standard face-to-face physiotherapy for 12 weeks. Assessments were performed at baseline, 12 weeks, and 24 weeks postoperatively. The primary outcomes were 2 subscales of the Foot and Ankle Ability Measure, with a noninferiority margin of 8 points for Foot and Ankle Ability Measure—Activities of Daily Living (FAAM-ADL) and 9 points for Foot and Ankle Ability Measure—Sports (FAAM-S). Secondary outcomes included balance tests (time-in-balance, foot-lift, and star excursion balance), functional tests (ankle dorsiflexion range of motion, side-hop, and figure-8 hop), and quality of life (FAAM questionnaire). We also collected intervention costs to evaluate cost-effectiveness. Statistical analyses included between-group comparisons of outcomes and nonparametric bootstrapping to calculate incremental cost-effectiveness ratios.

**Results:**

Baseline characteristics were similar between groups (except for a difference in foot-lift test performance). By the 24-week follow-up, improvements in the primary outcomes were comparable between the DT and physiotherapy groups, with adjusted between-group differences of 0.36 (95% CI −1.01 to 1.72) for FAAM-ADL and 1.67 (95% CI −0.61 to 3.96) for FAAM-S. These differences fell within the predefined noninferiority margins, demonstrating noninferiority of the digital program. No significant between-group differences were observed in secondary outcomes (all *P*>.05). The average cost per patient was lower in the DT group (CNY 53,551; an exchange rate of US $1=CNY 7 was applied) than in the physiotherapy group (CNY 59,372), yielding an incremental cost of CNY −14,451 in favor of the digital intervention. The bootstrapped incremental cost-effectiveness ratios were CNY −16,396 for FAAM-ADL and CNY −114,131 for FAAM-S, indicating that DT was more cost-effective.

**Conclusions:**

Individually tailored PAST delivered via a DT system was found to be clinically noninferior to conventional face-to-face physiotherapy and more cost-effective, supporting its use as a viable rehabilitation alternative for CAI patients after MBS.

## Introduction

Sports participation often leads to ankle sprains, one of the most common musculoskeletal injuries in physically active individuals [1,2]. In the United States, over 1,000,000 ankle sprains are treated in emergency departments each year, incurring substantial health care costs [3]. These costs translate into billions annually; for example, one systematic review estimated about US $6.2 billion in health care expenditures for ankle injuries among US high school athletes [2]. In addition to direct medical costs, ankle sprains also impose indirect economic burdens: roughly 25% of injured individuals are unable to attend work or school for at least a week following the injury [3]. Despite this burden, athletes sustaining lateral ankle sprains have a high reinjury rate (70%‐80%), which can lead to chronic ankle instability (CAI). After lateral ankle sprains, clinicians must address factors such as muscle weakness, impaired postural control, limited range of motion, and recurrent injury to restore function [4]. Thus, effective strategies are needed to improve ankle stability and prevent CAI in this high-risk population.

CAI is characterized by persistent lateral ankle instability with recurrent sprains [5]. This condition involves both ligamentous laxity and sensorimotor impairments: patients with CAI often exhibit deficits in postural control and muscle strength, in addition to structural laxity of the ankle joint [6-8]. Surgical intervention, such as the modified Brostrom surgery (MBS), is a well-accepted method to restore mechanical stability [9]. On the other hand, effective rehabilitation postsurgery is now shifting toward a more functional approach, emphasizing dynamic, closed kinetic-chain activity instead of quiet standing and open kinetic-chain positioning. However, specialized physiotherapists cannot afford to serve this patient group in China. Since the growing rehabilitation needs of adolescents after CAI cannot be met by the current labor force of physiotherapists in China, the exploration for new effective alternatives to ensure reliable and accessible postoperative physical rehabilitation is vital and urgent [10].

An alternative approach to treatment is using digital training (DT) systems to administer training programs to patients in their own homes. This method may offer a solution to the challenges of accessibility faced by patients residing in remote or rural regions, as well as those in urban areas with limited transportation options [11,12]. Following their discharge from the hospital, numerous patients who have undergone MBS encounter difficulties accessing health care services. Patients residing in rural regions encounter additional barriers to access due to the lengthy travel times and distances involved for both patients and health care providers. The adoption of technology-enabled home exercise programs could incentivize patients to engage in exercise routines more frequently, potentially rectifying the strength impairments often observed in CAI. In addition to resolving accessibility challenges, implementing such home exercise programs could also lead to cost savings in the provision of health care services.

Recent studies have explored home-based and telerehabilitation for CAI. For example, both center-based and home-based exercise programs over 6 weeks significantly improved strength, balance, and function in athletes with CAI, although the center-based program yielded somewhat greater gains in certain functional tests [13]. Similarly, Yang et al [14] found that a 4-week telerehabilitation exercise program produced functional improvements comparable to those from clinic-based therapy in CAI patients. These findings suggest that technology-assisted, home-based rehabilitation can be effective. However, previous work has largely focused on nonsurgical CAI populations and has not included economic analyses. Critically, no high-quality randomized trial has yet tested a cost-effective, individually tailored rehabilitation program for patients recovering from surgical treatment of CAI.

Several studies have explored the use of digital-based training programs in postoperative rehabilitation programs for total knee arthroplasty and total hip arthroplasty, demonstrating promising outcomes [15]. Not only have these programs been found to produce comparable results to traditional in-person rehabilitation, but the majority of patients enrolled in digital-based training programs have expressed satisfaction with this alternative approach [15]. The goal of this study is to determine whether an individually tailored physiotherapeutic ankle-specific training (PAST) program via a DT system has similar clinical outcomes and cost-effectiveness as a conventional face-to-face training program for patients with CAI following MBS. We hypothesize that patients with CAI who undergo a structured home-based rehabilitation training program will experience noninferior ankle stability and function and incur lower ankle-related medical expenses compared to those receiving conventional face-to-face training by the physiotherapist.

## Methods

### Study Design and Setting

A single trauma center, randomized, single-blinded, 2-armed, noninferiority clinical trial was conducted to compare DT system group (DT group) with conventional physiotherapist face-to-face training group (physiotherapy group) from January 2022 to January 2024 in a single trauma center.

The study was registered in the Chinese Clinical Trial Registry (ChiCTR2300075292), and all procedures were conducted in accordance with the Declaration of Helsinki. All participants signed a statement of informed consent after receiving clarifications regarding the study objectives and procedures. The trial protocol is provided in Multimedia Appendix 1 [1,4-12,15-39]. The study followed the Consolidated Standards of Reporting Trials (CONSORT) reporting guideline.

All participants were enrolled from the surgical waiting lists of podiatrists in Huashan Hospital. A total of 2 physicians (XG and XW) screened patients to determine their eligibility based on the inclusion criteria and observed or followed them up until the conclusion of the study. Once a physiotherapist noticed that a patient had not used the DT system during the study period, they would promptly inquire about the reasons for the patient’s failure to engage in the PAST program. Patients were enrolled in the study after obtaining informed consent, and baseline evaluations for all participants were performed by a single physiotherapist. Randomization was performed on the first day after surgery by the study coordinator. Additionally, 2 independent physiotherapists made contact with participants to proceed with allocation and intervention. All participants received a 12-week intervention and were followed for 24 weeks. Participants were evaluated at baseline (E1, before surgery), at 12 weeks after surgery (E2, at the end of intervention), and 24 weeks after surgery (E3) by independent evaluators blinded to the staff of enrollment. The functional outcome measures were collected by two physiotherapists in outpatient clinics. The self-assessed outcome measures were also completed in an outpatient clinic using online questionnaires, assisted by these 2 physiotherapists, who were blinded to group allocation.

### Ethical Considerations

This study was approved by the Research Ethics Committee of Huashan Hospital, Fudan University (IRB 2022‐069), and all procedures were conducted in accordance with the Declaration of Helsinki and local institutional guidelines. Written informed consent was obtained from all participants before enrollment. To ensure privacy and confidentiality, all collected data were coded (deidentified) and securely stored using the hospital’s electronic data capture system on local servers, accessible only to the research team. No financial compensation or other incentives were provided to participants for their involvement in this study.

### Participants

Participants had to meet the eligibility criteria (Textbox 1).

Textbox 1. **Eligibility criteria.**

Inclusion criteria:

1. Awaiting modified Brostrom surgery following a diagnosis of chronic ankle instability.
2. Discharged from the hospital and returned home.
3. Residing in an area with access to high-speed internet services (with a minimum upload speed of 512 kb/s).
4. Living within a 1-hour driving distance from the treating hospital.

Exclusion criteria:

1. Having any of the following health conditions that could interfere with the tests or the rehabilitation program:
  - Undergoing other lower-limb surgeries within the past 9 months.
  - Concomitant lower-extremity injuries, neurological disorders (eg, stroke, peripheral neuropathy, and multiple sclerosis).
  - Severe cardiopulmonary disease
  - Balance or vestibular disorders unrelated to the ankle.
  - Musculoskeletal disorders affecting other regions (eg, chronic low back pain and upper limb dysfunction).
  - Uncontrolled metabolic or systemic diseases (eg, poorly controlled diabetes and rheumatoid arthritis).
2. Planning a second lower-limb surgery within the next 4 months.
3. Experiencing cognitive or collaboration problems.
4. Encountering major postoperative complications.
5. If they withdrew from the study or failed to engage in 75% of the entire training regimen.

### Intervention and Control Conditions

#### Overview

The individually tailored PAST program was based on the recommendations of a group of experts [16-18], including 3‐4 sessions per week of 45 minutes to 60 minutes; the intensity and duration were an assessment by the supervising physiotherapist according to each patient’s tolerance and needs (for details, see Multimedia Appendix 2). Advice concerning pain control, walking aids, and the return to activities was also given to the patients. The difficulty and intensity of the exercise were increased according to each patient’s tolerance and needs. Our research team involved 2 two senior physiotherapists (YL and XZ), both having over 5 years of experience in the postoperative training of CAI.

#### DT Group

The PAST program was delivered via a smartphone-based digital rehabilitation platform called JoyMotion (Shanghai Medmotion Medical Management Co Ltd). JoyMotion is a mobile app available for free on the Apple App Store in China. It provides patients with daily guided ankle exercise routines, complete with detailed instructions and illustrative exercise images and videos, and it enables interactive telerehabilitation features. A technician assisted each patient in installing the JoyMotion app on their device before hospital discharge, ensuring the patient could comfortably use it at home via their own WiFi network. The supervising physiotherapist scheduled weekly live video call sessions through the app (2-way video and audio) at prearranged times to monitor progress, provide feedback, and adjust the exercise regimen as needed. The rehabilitation exercises were individualized by the physiotherapist and assigned to the patient as daily “tasks” within the app, which patients accessed using their personal accounts. JoyMotion automatically tracked each patient’s exercise performance and completion rate and provided feedback on their training progress.

Importantly, JoyMotion also incorporated an adherence-monitoring system: if a patient failed to complete a scheduled exercise session, the app would send an automated reminder through its chat feature to prompt timely completion. If the patient continued to miss exercises over a period of time, the system would alert the physiotherapist, who then reached out to the patient (eg, via phone call or message) to ensure compliance and address any issues. Finally, if a patient experienced any difficulties or physical discomfort during the training program, the physiotherapist could consult an orthopedic physician for additional medical support (the physiotherapy control group’s intervention was described as standard face-to-face outpatient physiotherapy sessions 3‐4 times per week, with similar exercise content and home-exercise assignments, supervised through in-person visits and patient-reported logs).

#### Physiotherapy Group

Patients in the physiotherapy group visited the appointed outpatient clinics 3 to 4 times each week for 12 weeks after discharge. Patients received conventional face-to-face training by the physiotherapist. Each patient in the physiotherapy group was under the care of the same dedicated physiotherapist for all sessions during the 12-week program to ensure continuity of care. The components of the face-to-face intervention and the following home exercises were prescribed following the PAST program and the physiotherapy group’s assessment before and after exercise. For the supervision of the physiotherapy group, physiotherapists recorded the times of participants’ visits and instructed patients to record how much homework they completed after outpatient clinic visits.

### Outcomes and Data Collection

#### Primary Outcomes

We used the Foot and Ankle Ability Measure (FAAM) questionnaire (original English version) to evaluate participants’ self-reported foot and ankle function. The FAAM is a tool used for evaluating the overall self-reported functional status in patients with musculoskeletal injuries and disorders affecting the leg, ankle, and foot [19]. This measure comprises 2 subscales, namely the Foot and Ankle Ability Measure—Activities of Daily Living (FAAM-ADL) and the Foot and Ankle Ability Measure—Sport (FAAM-S), both of which are scored on a 0% to 100% scale, where a higher score indicates a better functional status [19]. The FAAM is considered a reliable questionnaire for assessing the function of adult patients with CAI [20]. The reported Minimal Detectable Change (MDC) scores for FAAM-ADL and FAAM-S are 3.9% to 4.8% and 7.6% to 7.9%, respectively [21]. Moreover, the minimal clinically important difference (MCID) scores for an adult population receiving treatment for musculoskeletal disorders in the leg, ankle, or foot are 8 and 9 points for FAAM-ADL and FAAM-S, respectively [19].

#### Secondary Outcomes

##### Time-in-Balance Test

Participants assumed a natural standing posture with their hands on their hips and eyes closed. They were directed to maintain their balance on the designated limb while the examiner timed the duration of the stance in seconds. Each attempt was limited to a maximum of 60 seconds, and any movement of the testing foot or contact with the contralateral foot resulted in termination of the trial. Before the test trials, a practice trial was allowed for the patient to become familiar with the procedure. The test was repeated 3 times, with 30 seconds of rest provided between each trial, and the duration of the longest trial was analyzed. This test has been shown to be reliable and sensitive to rehabilitation (intraclass correlation coefficient [ICC]=0.99), and the methodology was consistent with previous reports [22,23].

##### Foot-Lift Test

Patients were instructed to stand on one foot, with their hands on their hips and their eyes closed, while maintaining an erect stance. During the 30-second test, the number of foot lifts was recorded, with any part of the foot leaving the floor considered a foot lift. Touching the floor with the contralateral foot was deemed an error. Patients were instructed to avoid removing their hands from their hips, opening their eyes, and touching the stance limb with the contralateral foot, but such actions were not regarded as errors. Before the test trials, a practice trial was permitted for patient familiarization. The test was conducted 3 times, with a 30-second rest between each trial, and the average number of foot lifts from the 3 trials was used for analysis. This test has been shown to be valid and responsive to rehabilitation (ICC=0.99), and the methodology is consistent with previous reports [22,23].

##### Star Excursion Balance Test

During the star excursion balance test, patients stood on their test limb and extended their reach as far as possible in each of 5 directions while maintaining their balance. The 5 directions evaluated were anterior, anteromedial, medial, posteromedial, and posterolateral. Each reach was made over a cloth tape measure securely attached to the floor, with the distance measured by the investigator in centimeters and normalized to the patient’s nontest limb length. Patients were given 4 practice trials in each direction with a 5-minute rest before the test sessions [23]. The test was conducted 3 times in each direction, with a 10-second rest between trials. The average of the 3 trials for each direction was used for analysis. This test has been demonstrated to be valid and responsive to rehabilitation (ICC range 0.81‐0.93), and the methodology is consistent with previous reports [24-26].

##### Ankle Dorsiflexion Range of Motion

A goniometer was used for measuring the ankle-dorsiflexion range of motion. The stationary arm of the goniometer is aligned with the fibula, the axis is placed at the lateral malleolus, and the moving arm follows the fifth metatarsal. Goniometry has ICC values around 0.80 to 0.90, indicating good reliability [27].

##### Side-Hop Test

During the lateral hop test, patients hopped laterally 30 cm on their involved limb for 10 repetitions as quickly as possible. The time taken to complete the test was recorded to the nearest 0.01 second using a handheld stopwatch (model AX725 Pro Memory; Accusplit). Patients were given a single practice trial for familiarization before the test trials. The test was conducted twice on the involved limb, with a 60-second rest provided between trials. The shortest trial was used for analysis. This test has been shown to be valid and responsive to rehabilitation (ICC=0.99), and the methodology is consistent with previous reports [22,28].

##### Figure-8 Hop Test

During the figure-8 hop test, patients hopped over a 5-meter distance on their test limb in a figure-8 pattern, and the time taken to complete the test was recorded to the nearest 0.01 second using a handheld stopwatch. Patients were given a single practice trial for familiarization before the test trials. The test was conducted twice on the involved limb, with a 60-second rest provided between trials. The shortest trial was used for analysis. This test has been shown to be valid and responsive to rehabilitation (ICC=0.98), and the methodology is consistent with previous reports [22,28].

##### Cost Measures

Costs assessed in this study included intervention costs, other health care expenses, costs for paid and informal home care, as well as expenses related to work absenteeism, presenteeism, and lost productivity in unpaid tasks.

Intervention costs were gathered from the hospital information system of Huashan Hospital and the online payment system of Shanghai Medmotion Medical Management Company. Additional health care expenses encompassed costs for primary health care (eg, general practitioner visits), secondary health care (eg, noninitial hospital visits), and both prescribed and over-the-counter medications, all of which were obtained from the hospital information system.

Paid home care expenses were evaluated by participants’ reports on the number of hours of paid care received, priced through direct inquiries at the 12-week and 24-week follow-ups. Informal care costs were derived from the total hours of assistance provided by family, friends, and volunteers, as reported by patients during outpatient follow-up visits. These costs were calculated by multiplying the total hours by the average hourly income in Shanghai.

Absenteeism and presenteeism costs were estimated using the Productivity Cost Questionnaire [29]. Absenteeism costs were determined by counting the number of sick days and valuing them through the Friction Cost Approach (FCA; friction period=24 weeks) with gender-specific price weights [17]. Presenteeism costs were assessed by having participants rate their performance level on days they worked despite health issues, on a scale from 0 (completely incapacitated) to 10 (fully functional). The cost of presenteeism was then calculated using the formula: Presenteeism days = ([10–performance level] ÷ 10) × number of days with health complaints and valued by gender-specific price weights [18].

Finally, costs for unpaid productivity losses were estimated by having participants report hours lost in performing unpaid tasks (eg, chores, volunteer work, and educational activities), which were then valued using the average hourly income in Shanghai [30].

### Adherence and Acceptability

Adherence was measured using several indicators: the number of calls received, the attendance rate of control group participants at sessions, and the experimental group participants’ reported completion of sessions [31]. Additionally, both groups rated their agreement with statements concerning adherence and acceptability on a scale from 0 (“strongly disagree”) to 10 (“strongly agree”). Participants also provided qualitative assessments of their perceptions of the exercise protocol’s outcomes [31].

Participants were recruited between January 2022 and mid-2023, and final follow-up assessments were completed in January 2024. The timeline for data collection of this study is shown in Multimedia Appendix 3.

### Sample Size, Randomization, and Blinding

Sample size was calculated based on the data of primary outcomes (FAAM-ADL and FAAM-S), by means of the noninferiority power calculation described by Jones et al [32].

The subscales of FAAM were calculated separately for the sample size. The MCID values determined by Paulsen et al [33] were used for the noninferiority margin (8 and 9 points for FAAM-ADL and FAAM-S). The intervention will be accepted as equivalent if the difference between the 2 groups is less than the MCID. Calculations were based on 80% power and a type I error of 5% (α=.05). The values of FAAM-ADL yielded the largest sample size of 30 per group; thus, we set a sample size of 40 per group based on a 25% dropout rate.

A computer-generated randomization list (SAS Proc Plan, SAS/STAT 9.3) was prepared by an independent statistician using a 1:1 permuted-block scheme (maximum block size 6). Allocation sequences were placed in sequentially numbered, opaque, sealed envelopes to ensure concealment. On the first postoperative day, the study coordinator (not involved in sequence generation) enrolled each patient and opened the next envelope to assign the intervention.

Outcome assessors and data analysts were blinded to group allocation for the entire study. Specifically, the physiotherapists collecting 12- and 24-week outcome measures were unaware of each participant’s group. Participants and treating therapists were not blinded due to the nature of the interventions, but data analyses were performed with group codes concealed.

### Statistical Analysis

Patient data were coded and securely stored using the hospital’s electronic data capture system, hosted on local servers. The primary analysis focused on the intention-to-treat (ITT) population, and the per-protocol population was used for sensitivity analysis; multiple imputation was used to handle missing follow-up data. The missing data mechanism was assessed by comparing baseline characteristics between participants with complete and incomplete follow-up data. No significant differences were found in age, sex, baseline functional scores, or other characteristics, supporting the assumption that data were missing completely at random. Given the low proportion of missing data (6/84, 7.1%) and this assessment, multiple imputation was considered appropriate.

Following treatment, continuous outcomes, including questionnaire scores and functional test results, were analyzed as changes from baseline. Outcome analyses were conducted using linear mixed-effects models to account for the repeated measurements (baseline, 12 weeks, and 24 weeks) from each patient. The model included treatment group, time, and their interaction as the primary fixed factors of interest. To adjust for potential confounding and increase precision, the following baseline covariates were also included: age, sex, BMI, manual worker, nonsteroidal anti-inflammatory drug use, and walking aid use. The model incorporated random intercepts for patients to capture the within-subject correlation over time.

Baseline scores for all functional outcomes were included as covariates to adjust for any initial differences. Treatment effects were estimated using least squares means. For the primary noninferiority assessment, the DT intervention was considered noninferior if the between-group differences and their 1-sided 95% CIs were within the predefined margins of 8 and 9 points for FAAM-ADL and FAAM-S, respectively, at the 24-week follow-up.

Statistical analyses were performed using Stata (version 17.0; StataCorp). All tests were 2-sided with a significance level of α=.05, except for the 1-sided noninferiority tests for the primary outcomes.

The incremental cost-effectiveness ratio (ICER) was used to assess the cost-effectiveness of DT compared with physiotherapy. The ICER is the difference in costs and outcomes between the DT and physiotherapy. The numerator in the cost-effectiveness ratio is the monetary cost of the DT intervention minus the monetary cost of physiotherapy. The annual costs of the projects were calculated by converting the 24-week costs, the period used for implementation. The denominator is the FAAM-ADL and FAAM-S usability gained by DT minus the FAAM-ADL and FAAM-S usability gained by the physiotherapy group. Bootstrapping was used for a pair-wise comparison of the mean costs and effects between the DT and physiotherapy groups. CIs for the mean differences in effects were obtained by bootstrapping (1000 replications). The bootstrapped cost and effect pairs were also graphically represented on a cost-effectiveness plane [34].

## Results

### Participants

**Figure 1.**
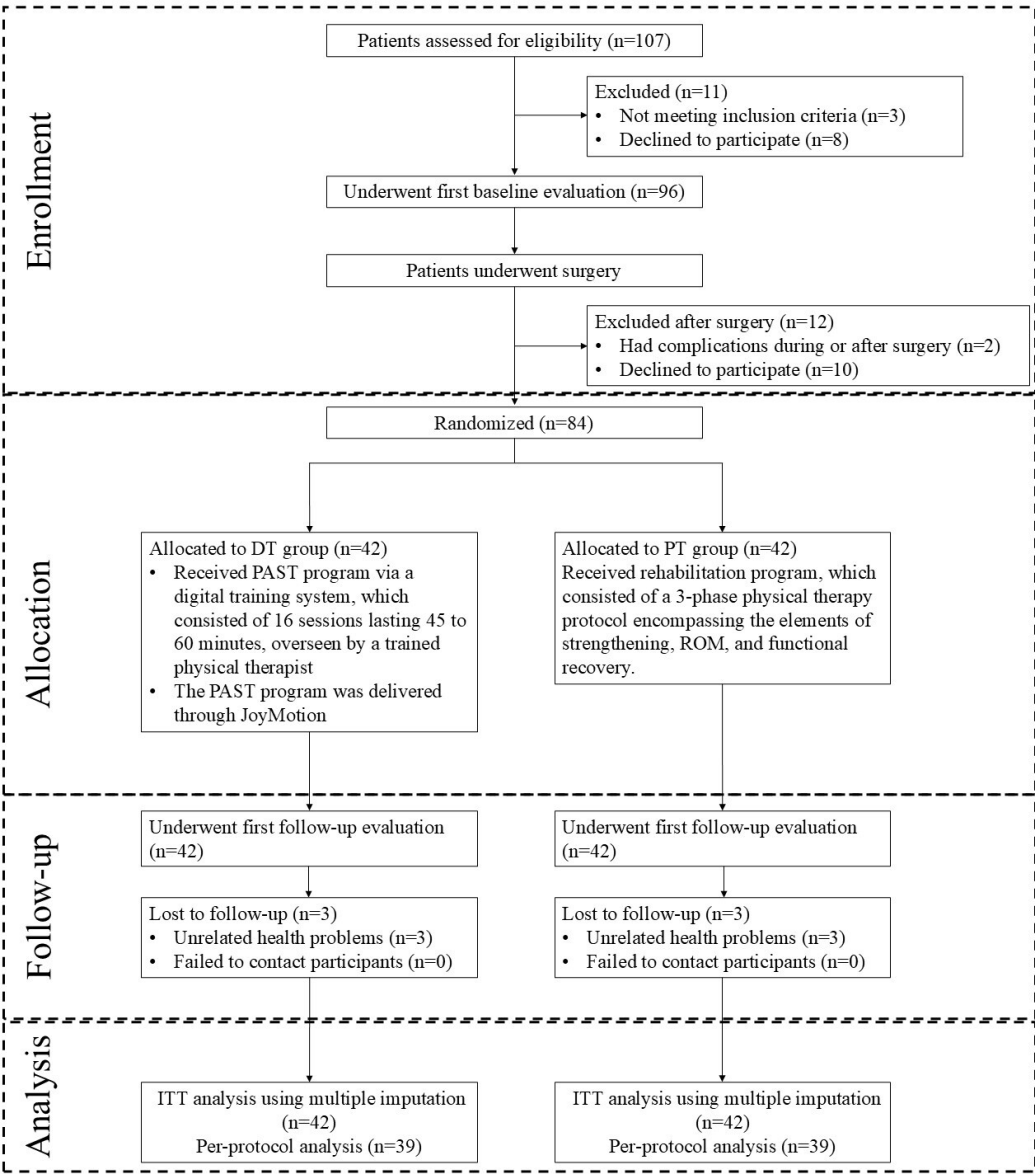
CONSORT (Consolidated Standards of Reporting Trials) flow diagram illustrating participant progress through each phase of the trial (enrollment, allocation, intervention, follow-up, and analysis). A total of 107 patients were assessed for eligibility, of whom 11 were excluded (3 did not meet the inclusion criteria and 8 declined to participate). A total of 96 patients underwent baseline evaluation and modified Broström surgery, after which 12 were excluded (2 due to surgical complications and 10 declined further participation). The remaining 84 patients were randomized into either the digital training (DT) group (n=42) or the physiotherapy group (n=42). All participants received their allocated intervention (DT group: 12-week digital PAST program and PT group: 12-week standard face-to-face physiotherapy) and completed the first follow-up at 12 weeks. By the 24-week follow-up, 3 patients in each group were lost to follow-up (all due to unrelated health problems), leaving 39 per group who completed the study. All 84 randomized patients were included in the intention-to-treat (ITT) analysis with missing data handled by multiple imputation, while 78 patients (39 in each group) were analyzed per protocol. DT: digital training group; PT: physiotherapy group; PAST: physiotherapeutic ankle-specific training; ROM: range of motion; ITT: intention-to-treat.

A total of 107 patients were screened, and 84 underwent surgery and were randomized (42 per group). Additionally, 3 patients in each group were lost to follow-up before 24 weeks (due to unrelated health issues), leaving 39 patients in each group who completed the study (Figure 1). All 84 randomized patients were included in the primary ITT analysis, and 78 patients (39 per group) who completed the protocol were included in the per-protocol analysis. Demographic data were specified in Table 1. The baseline characteristics of the DT and physiotherapy groups were compared. The average ages were similar, with 45.9 years in the DT group and 45 years in the physiotherapy group (*P*=.66). The proportion of male patients was almost the same, with 20/42 participants (48%) in the DT group and 21/42 (50%) in the physiotherapy group (*P*=.83). The DT group had a higher BMI (24.57 kg/m²) than the physiotherapy group (23.21 kg/m²; *P*=.03). Both groups had a similar distribution of manual workers (*P*=.83), but more patients in the physiotherapy group had a higher education level (*P*=.12). Insurance type was nearly the same for both groups (*P*=.95).

**Table 1.** Baseline characteristics of the digital training group and physiotherapy group (intention-to-treat population).

Sample characteristic	DT^a^^,^^b^ group (n=42)	PT^c^^,^^d^ group (n=42)	*P* value
Age (years), mean (SD)	45.90 (9.33)	45.00 (9.37)	.66
Male patients, n (%)	20 (47.62)	21 (50)	.83
BMI (kg/m^2^), mean (SD)	24.57 (2.81)	23.21 (2.64)	.03
Occupation, n (%)			.83
Manual worker	24 (57.14)	23 (54.76)	
Nonmanual worker	18 (42.86)	19 (45.24)	
Education, n (%)			.12
Lower than high school	21 (50)	14 (33.33)	
High school and above	21 (50)	28 (66.67)	
Insurance type, n (%)			.95
Government	34 (80.95)	35 (83.33)	
Commercial	2 (4.76)	2 (4.76)	
Out-of-pocket	6 (14.29)	5 (11.9)	
Paracetamol and NSAID^e^, n (%)	20 (47.62)	26 (61.9)	.19
Walking aid, n (%)	2 (4.76)	2 (4.76)	>.99
PROM^f^, mean (SD)			
FAAM-ADL^g^	87.26 (2.33)	86.98 (1.47)	.50
FAAM-S^h^	69.57 (4.16)	70.31 (2.55)	.33
Time-in-balance test (s)	19.59 (3.10)	18.78 (3.12)	.24
Foot-lift test (times)	5.43 (1.23)	4.57 (1.13)	.001
Star excursion balance test, mean (SD)			
Anterior (cm)	72.91 (1.88)	73.18 (1.91)	.52
Posteromedial (cm)	81.48 (3.16)	82.23 (3.84)	.33
Posterolateral (cm)	73.87 (3.61)	74.16 (4.02)	.72
Function, mean (SD)			
Ankle-dorsiflexion range of motion (°)	8.38 (0.58)	8.62 (0.49)	.046
Side-hop test (s)	13.84 (1.02)	13.68 (0.99)	.46
Figure-8 hop test (s)	15.40 (0.70)	15.20 (0.63)	.18

a DT: digital training group.

b Individually tailored physiotherapeutic ankle-specific training via a digital training system.

c PT: physiotherapy group.

d Conventional physiotherapist face-to-face training.

e NSAID: nonsteroidal anti-inflammatory drug.

f PROM: patients reported outcome measures.

g FAAM-ADL: Foot and Ankle Ability Measure—Activities of Daily Living.

h FAAM-S: Foot and Ankle Ability Measure—Sport.

In terms of medication, 20/42 participants (48%) in the DT group and 26/42 (62%) in the physiotherapy group used paracetamol and nonsteroidal anti-inflammatory drugs (*P*=.19). A small number of participants in each group used walking aids (2/42, 5%). PROM scores like FAAM-ADL and FAAM-S, and most functional test scores, showed no significant differences. However, the DT group performed better in the foot-lift test (*P*=.001). The ankle-dorsiflexion range of motion was slightly less in the DT group (*P*=.05). Other tests, like the side-hop test and figure-8 hop test, were similar in both groups.

### Primary and Secondary Outcomes

At the 24-week follow-up evaluation, the mean differences between the groups with regard to the FAAM-ADL and FAAM-S, adjusted for baseline values, were near zero (for 84 patients in the ITT analysis): 0.36 (95% CI −1.01 to 1.72) for FAAM-ADL and 1.67 (95% CI −0.61 to 3.96) for FAAM-S (Table 2). The CIs were all within the predetermined zone of noninferiority (8 and 9 points for FAAM-ADL and FAAM-S). Additionally, the time-in-balance test, the star excursion balance test-posteromedial, ankle-dorsiflexion range of motion, and side-hop test demonstrated nonsignificant differences between the 2 groups at 24-week follow-up (Table 3). The mean differences between the groups with regard to other secondary outcomes, adjusted for baseline values, were −0.92 (95% CI −1.29 to −0.54) for the foot-lift test, −0.97 (95% CI −1.48 to −0.46) for star excursion balance test-anterior, −1.90 (95% CI −2.92 to −0.88) for star excursion balance test-posterolateral, and 1.0 (95% CI 0.83 to 1.17) for the figure-8 hop test (Table 3).

**Table 2.** Changes in outcomes for the digital training and physiotherapy groups at weeks 12 and 24 follow-up, intention-to-treat analysis using multiple imputation.^a^

Outcome	12 weeks follow-up			24 weeks follow-up		
	DT^b^^,^^c^ group (n=42), mean (SD)	Physiotherapy group^d^ (n=42), mean (SD)	*P* value	DT group (n=42), mean (SD)	Physiotherapy group^d^ (n=42), mean (SD)	*P* value
PROMS^e^						
FAAM-ADL^f^	8.83 (3.33)	8.40 (3.16)	.55	9.33 (3.62)	8.98 (4.42)	.69
FAAM-S^g^	12.48 (3.49)	12.90 (1.10)	.45	18.27 (7.07)	16.60 (5.90)	.24
Time-in-balance test (s)	15.15 (1.78)	16.83 (1.54)	<.001	26.11 (2.21)	25.88 (4.68)	.77
Foot-lift test (times)	3.07 (1.05)	3.83 (0.85)	<.001	3.75 (1.08)	4.67 (0.90)	<.001
Star excursion balance test						
Anterior (cm)	6.51 (1.13)	7.33 (0.82)	<.001	9.26 (1.49)	10.23 (1.59)	.005
Posteromedial (cm)	10.82 (1.97)	10.49 (3.89)	.63	13.52 (2.55)	12.76 (4.35)	.33
Posterolateral (cm)	11.25 (2.76)	12.75 (2.66)	.01	14.48 (3.01)	16.38 (3.07)	.005
Function						
Ankle-dorsiflexion range of motion (°)	2.31 (0.47)	2.55 (0.50)	.03	3.35 (0.57)	3.29 (0.49)	.61
Side-hop test (s)	–4.30 (0.79)	–4.17 (0.70)	.40	–5.55 (0.87)	–5.32 (0.95)	.24
Figure-8 hop test (s)	–1.80 (0.43)	–2.65 (0.38)	<.001	–2.90 (0.55)	–3.90 (0.46)	<.001

a Values represent the mean change from baseline for each group, reported as mean (SD), with *P* values comparing between-group differences at each follow-up point. Functional outcomes include primary endpoints (Foot and Ankle Ability Measure—Activities of Daily Living and Foot and Ankle Ability Measure—Sports subscale) and secondary performance-based tests.

b DT: digital training.

c Individually tailored physiotherapeutic ankle-specific training via a digital training system.

d Conventional physiotherapist face-to-face training.

e PROM: patient-reported outcome measure.

f FAAM-ADL: Foot and Ankle Ability Measure—Activities of Daily Living.

g FAAM-S: Foot and Ankle Ability Measure—Sport.

**Table 3.** Effectiveness estimates from linear mixed effects models (applied intention-to-treat analysis using multiple imputation).

Outcome^a^	12 weeks follow-up			24 weeks follow-up		
	Coefficient^b^ (95% CI)		*P* value	Coefficient^b^ (95% CI)		*P* value
PROMs^c^						
FAAM-ADL^d^	0.429 (−0.94 to 1.79)		.54	0.355 (−1.01 to 1.72)		.61
FAAM-S^e^	−0.429 (−2.71 to 1.86)		.71	1.672 (−0.61 to 3.96)		.15
Time-in-balance test (s)	−1.679 (−2.97 to −0.39)		.01	0.230 (−1.06 to 1.52)		.73
Foot-lift test (times)	−0.762 (−1.13 to −0.39)		<.001	−0.915 (−1.29 to −0.54)		<.001
Star excursion balance test						
Anterior (cm)	−0.819 (−1.33 to −0.31)		.002	−0.970 (−1.48 to −0.46)		<.001
Posteromedial (cm)	0.331 (−0.85 to 1.51)		.58	0.758 (−0.42 to 1.94)		.21
Posterolateral (cm)	−1.498 (−2.52 to −0.48)		.004	−1.897 (−2.92 to −0.88)		<.001
Function						
Ankle-dorsiflexion range of motion (°)	−0.238 (−0.44 to −0.04)		.02	0.059 (−0.14 to 0.26)		.56
Side-hop test (s)	−0.138 (−0.44 to 0.16)		.37	−0.235 (−0.53 to 0.06)		.12
Figure-8 hop test (s)	0.840 (0.67 to 1.01)		<.001	0.999 (0.83 to 1.17)		<.001

a All outcome measures were adjusted for baseline values in the model.

b Each coefficient represents the estimated between-group difference in the change from baseline (digital training [DT] group−physiotherapy group) for the specified outcome at that follow-up time point. Positive coefficients indicate higher scores in the DT group compared to the physiotherapy group, whereas negative values indicate lower scores in the DT group. Each estimate is presented with its 95% CI and corresponding *P* value.

c PROM: patient-reported outcome measure.

d FAAM-ADL: Foot and Ankle Ability Measure—Activities of Daily Living.

e FAAM-S: Foot and Ankle Ability Measure—Sport.

In sensitivity analysis by using the per-protocol population (78 patients), the mean differences between the groups with regard to the FAAM-ADL and FAAM-S, adjusted for baseline values, were near zero: 0.05 (95% CI −1.30 to 1.40) for FAAM-ADL and 0.59 (95% CI −1.52 to 2.70) for FAAM-S (MultimediaAppendices 4  5).

Values represent the mean change from baseline for each group, reported as mean (SD), with *P* values comparing between-group differences at each follow-up point. Functional outcomes include primary endpoints (FAAM-ADL and FAAM-S subscales) and secondary performance-based tests.

Each coefficient represents the estimated between-group difference in the change from baseline (DT group minus physiotherapy group) for the specified outcome at that follow-up time point. Positive coefficients indicate higher scores in the DT group compared to the PT group, whereas negative values indicate lower scores in the DT group. Each estimate is presented with its 95% CI value and corresponding *P* value. All outcome measures were adjusted for baseline values in the model.

### Cost-Effectiveness Outcomes

The average total cost per patient over 24 weeks was lower in the DT group (CNY 53,551.36; the exchange rate is US $1=CNY 7) than in the physiotherapy group (CNY 59,372.03; see Table 4), though this difference was not statistically significant (*P*=.08). The DT group had significantly lower costs in several categories, including the physical therapist cost (*P*<.001), primary care (*P*<.001), secondary care (*P*=.005), paid home care (*P*=.01), medication (*P*=.004), and transportation (*P*=.01). Hospital stay and nutrition costs, as well as opportunity costs such as lost wages for patients and families, were not significantly different between the groups (Table 4).

**Table 4.** Average total cost per patient in the digital training and physiotherapy groups during the 24 weeks after the surgery (applied intention-to-treat analysis using multiple imputation).^a^

Cost^b^ category (CNY^c^)	DT^d^^,^^e^ group (n=42), mean (SD)	Physiotherapy group^f^ (n=42), mean (SD)	*P* value
Intervention cost			
Physical therapist cost	6980.00 (0.00)	11,146.15 (886.77)	<.001
Hospital stay cost	5779.46 (1145.74)	5367.82 (1275.95)	.14
Other medical cost			
Primary care	123.08 (45.66)	2064.10 (164.22)	<.001
Secondary care	1193.97 (967.16)	1960.26 (1334.55)	.005
Paid home care	214.36 (193.11)	351.69 (259.70)	.01
Medication	5359.90 (998.53)	6243.72 (1541.36)	.004
Nonmedical cost			
Transportation cost	616.87 (306.03)	909.08 (640.15)	.01
Nutrition cost	1881.36 (631.08)	1983.08 (646.06)	.48
Opportunity cost			
Lost wages for patients	28,027.36 (15,048.86)	27,069.31 (12,903.69)	.76
Lost wages for families	3375.00 (3306.83)	2276.82 (2951.27)	.13
Total cost	53,551.36 (15,776.85)	59,372.03 (12,507.48)	.08

a Values are reported as mean cost per patient in each category for the 24-week period, with *P* values for the between-group comparisons.

b Costs are categorized into intervention costs (eg, costs of the physiotherapist or digital platform use, and any hospital stay related to the rehabilitation), other medical costs (eg, primary care visits, secondary or specialist care visits, paid home care services, and medications), nonmedical costs (eg, transportation expenses for clinic visits and nutritional supplements), and opportunity costs (productivity losses such as lost wages for patients and family caregivers).

c CNY: Chinese yuan. US $1=CNY 7.

d DT: digital training.

e Individually tailored physiotherapeutic ankle-specific training via a digital training system.

f Conventional physiotherapist face-to-face training.

The DT group had a lower incremental cost than the physiotherapy group, with an average reduction of CNY 5820.67. The ICERs for the FAAM-ADL and FAAM-S scores were −16,396.25 and −3481.26, respectively, in the ITT analysis at 24 weeks (Table 5). These negative ICER values suggest that the DT group was more cost-effective, achieving similar or slightly better outcomes at a lower cost compared to the PT group.

DT =Individually tailored physiotherapeutic ankle-specific training (PAST) via a digital training system; PT =Conventional physiotherapist face-to-face training; CNY =Chinese Yuan

Costs are categorized into intervention costs (eg, costs of the physiotherapist or digital platform use, and any hospital stay related to the rehabilitation), other medical costs (eg, primary care visits, secondary or specialist care visits, paid home care services, and medications), non-medical costs (eg, transportation expenses for clinic visits and nutritional supplements), and opportunity costs (productivity losses such as lost wages for patients and family caregivers). Values are reported as mean cost per patient in each category for the 24-week period, with *P* values for the between-group comparisons.

CNY =ChineseYuan; ITT =Intention-to-treat; FAAM-ADL=TheFoot and Ankle Ability Measure-activities of daily living; FAAM-S=TheFoot and Ankle Ability Measure-sport

The table displays the incremental cost, incremental effect in primary outcome scores, and the resulting ICER. ICER is defined as the incremental cost divided by the incremental effect, representing the additional cost per unit improvement in outcome for the DT group versus the physiotherapy group. Incremental effects are given for both primary outcome measures, the FAAM-ADL score and FAAM-S score, which correspond to changes in the FAAM-ADL and FAAM-S subscales, respectively. ICER values are presented for the 24-week follow-up under both the ITT (with multiple imputation) and per-protocol analyses. A negative ICER indicates that the DT intervention achieved equivalent or better outcomes at a lower cost compared to physiotherapy.

**Table 5.** Incremental cost-effectiveness ratio (ICER) for primary outcomes.^a^

	Incremental cost, CNY^b^	Incremental FAAM-ADL^c^ score	Incremental FAAM-S^d^ score	ICER^e^ (FAAM-ADL)	ICER (FAAM-S)
ITT^f^ using multiple imputation (mixed effects; week 24)	−5820.67 (−12,241.61 to 600.27)	0.35 (−1.01 to 1.72)	1.67 (−0.61 to 3.96)	−16,396.25	−3481.26
Per protocol (mixed effects; week 24)	−5820.67 (−12,241.61 to 600.27)	0.05 (−1.30 to 1.40)	0.59 (−1.52 to 2.70)	−11,4130.78	−9865.54

a The table displays the incremental cost, incremental effect in primary outcome scores, and the resulting incremental cost-effectiveness ratio (ICER). ICER is defined as the incremental cost divided by the incremental effect, representing the additional cost per unit improvement in outcome for the digital training (DT) group versus the physiotherapy group. Incremental effects are given for both primary outcome measures, the FAAM-ADL score and FAAM-S score, which correspond to changes in the FAAM-ADL and FAAM-S subscales, respectively. ICER values are presented for the 24-week follow-up under both the intention-to-treat (with multiple imputation) and per-protocol analyses. A negative ICER indicates that the DT intervention achieved equivalent or better outcomes at a lower cost compared to physiotherapy.

b CNY: Chinese yuan. US $1=CNY 7.

c FAAM-ADL: Foot and Ankle Ability Measure—Activities of Daily Living.

d FAAM-S: Foot and Ankle Ability Measure—Sport.

e ICER: incremental cost-effectiveness ratio.

f ITT: intention-to-treat.

### Adherence and Acceptability

Phone calls with the DT group were largely conducted as scheduled. Adherence rates were high in both groups, with participants completing over 95% of all scheduled sessions (approximately 35 out of 36 sessions) in each group. The DT group averaged 3.8 sessions per week (SD 1.2), while the physiotherapy group averaged 3.6 sessions (SD 1.3). Additionally, participants in both groups reported positive perceptions of the treatment received, with average scores of 8.5 out of 10 for all evaluated aspects, as documented in Table 6. There was no significant difference between groups with regard to positive perceptions of the treatment received (Table 6).

**Table 6.** Patients’ adherence to treatment.

Outcome measure^a^	DT^b^^,^^c^ group (n=42), mean (SD)	Physiotherapy^d^ group (n=42), mean (SD)	*P* value
Number of sessions performed per week	3.8 (1.2)	3.6 (1.3)	<.001
Agreement with the following questions (from 0 to 10)			
To what extent did you agree to accept the allocated exercise plan?	8.4 (1.1)	8.6 (1.0)	.18
To what extent did you do the exercise program as recommended?	8.2 (1.3)	8.4 (1.9)	.98
To what extent do you agree that the intervention relieved your pain?	8.7 (1.6)	8.9 (1.3)	.89
To what extent do you agree that the intervention improved your function?	8.8 (1.7)	9.0 (0.8)	.10
To what extent were you satisfied with the exercise protocol?	9.5 (0.5)	9.4 (0.4)	.55

a Adherence is quantified as the average number of training sessions completed per week by each patient over the intervention period. Treatment acceptability was evaluated via patient self-reports on a series of statements about the exercise program’s acceptability and perceived benefits. For each statement, participants rated their level of agreement on a 0-10 scale, where 0 indicates “strongly disagree,” and 10 indicates “strongly agree.” The table presents the mean (SD) agreement scores for each question in each group, alongside *P* values for comparisons between groups. Higher scores reflect greater agreement or satisfaction with the aspect of treatment in question.

b DT: digital training.

c Individually tailored physiotherapeutic ankle-specific training via a digital training system.

d Conventional physiotherapist face-to-face training.

### Adverse Events

During the follow-up period, a similar proportion of participants in both groups reported adverse events. No serious events were related to the DT intervention, while a minor event was possibly related to the physiotherapy intervention (Table 7). The proportions of participants lost to follow-up were equivalent in both groups, with most losses occurring at the final follow-up survey (Figure 1).

**Table 7.** Adverse events and serious adverse events.

Events	DT^a^^,^^b^ group (n=42)	Physiotherapy^c^ group (n=42)
Adverse events		
Patients with adverse events, n (%)	4 (9.5)	5 (11.9)
Events related to study therapy, n	0	1^d^
Events unrelated to study therapy, n	5	5
Type of event, n		
Involved ankle		
Pain	2	2
Bruising	1	1
Swelling	0	1^d^
Signs of infection (swelling, redness, heat, or pus)	0	0
Mobilization under anesthesia	0	0
Other		
Fall with minor symptoms	1	0
Nausea and dizziness	0	0
Back pain	0	1
Anxiety about ankle recovery	1	1
Serious adverse events^e^		
Patients with serious adverse events, n (%)	3 (7.1)	3 (7.1)
Events related to study therapy, n	0	0
Events unrelated to study therapy, n	3	3
Type of event, n		
Death	0	0
Hospitalization	3	2
Degradation of the general condition	0	1^f^
Hip fracture due to fall	1^f^	0
Gastrointestinal disorder	0	1
Cardiac arrhythmia	1^e^	0
Spinal surgery	1^e^	1^e^

a DT: digital training.

b Individually tailored physiotherapeutic ankle-specific training via a digital training system.

c Conventional physiotherapist face-to-face training.

d A patient fell during intervention with minor consequent symptoms.

e Patients with serious adverse events were automatically withdrawn from the study.

f Events related to hospitalization.

## Discussion

### Principal Findings

This study aimed to compare the effectiveness and cost-efficiency of individually tailored PAST program via a DT system with conventional physiotherapist face-to-face training for patients with CAI following MBS. The consistently small intergroup differences and narrow CIs provide robust scientific evidence supporting the clinical noninferiority of the DT system and underscore its relevance for patient follow-up after CAI.

Orthopedic DT studies conducted previously were limited by short intervention periods (typically 2 weeks), simulations of home environments, or the need for assistants to help participants set up. In contrast, this study’s distinctive feature is the remote delivery of a 12-week intervention directly into the participants’ homes, without on-site technical support or requirements for high-quality internet connection or mobile data coverage. This makes the findings broadly applicable to the Chinese population. Additionally, the technology used in this study is readily available and commonly owned by the general public.

Over the 24-week follow-up period, both DT and physiotherapy groups showed significant improvements in functional outcomes, including patient-reported outcome measures like FAAM-ADL and FAAM-S. During both 12 and 24 weeks, the differences in FAAM-ADL and FAAM-S scores between the DT and physiotherapy groups were not statistically significant, indicating comparable effectiveness (*P*>.05 for all comparisons; see Table 2). Additionally, the changes in various functional tests, such as the time-in-balance test, foot-lift test, star excursion balance test, and the figure-8 hop test, showed that the physiotherapy group had slightly better performance in some respects, particularly at the 12-week mark. However, by 24 weeks, the differences between the groups had diminished, indicating that the DT system is capable of achieving noninferiority long-term outcomes compared to traditional physiotherapy interventions. These results are consistent with previous studies on DT systems for other diseases. Hughes et al [40] found improvements in Western Ontario and McMaster Universities Osteoarthritis Index pain, stiffness, and function scores of 23%, 17%, and 23%, respectively, at the end of an 8-week exercise and behavior-change program for osteoarthritis. Similarly, Hurley et al [41] demonstrated per protocol improvements in Western Ontario and McMaster Universities Osteoarthritis Index pain and function of 31% and 26%, respectively, at the end of a 6-week rehabilitation program combining self-management and exercise for chronic knee pain. It is important to acknowledge that the foot-lift test may have been influenced by a ceiling effect in our study. In participants with higher baseline balance ability (better function), performance on this single-leg stance test was already near optimal at baseline, leaving little room for improvement over time. Such ceiling effects can limit the test’s sensitivity to change. Indeed, many standard clinical balance measures show reduced ability to detect improvement in high-functioning individuals [42,43]. In line with this, static balance tests like the foot-lift test sometimes fail to distinguish performance differences in highly trained or less-impaired populations. In our study, the group with better baseline function achieved near-perfect scores on the foot elevation test, which likely masked potential improvements and contributed to the nonsignificant change observed. This limitation does not negate our findings, but it suggests that more challenging balance assessments might be necessary to fully capture improvements in postural control for patients who begin with relatively good balance.

A key finding of this study is the cost-effectiveness of the DT system compared to conventional physiotherapy training. The total cost per patient over the 24-week period was lower for the DT group (CNY 53,551.36) than for the physiotherapy group (CNY 59,372.03), though this difference was not statistically significant (*P*=.08). The cost savings in the DT group were mainly driven by significantly lower physical therapist costs (*P*<.001), primary and secondary care costs, medication costs, and transportation costs. The ICER analysis further supported the cost-effectiveness of the DT system, with negative ICERs for both FAAM-ADL and FAAM-S scores, indicating that the DT system is less costly and equally effective. Other DT programs for diseases like knee osteoarthritis have also demonstrated lower health care costs as compared to usual care [41].

No previous studies have examined a digital rehabilitation program specifically in post-Broström CAI patients. However, similar research in related contexts supports our findings. Yang et al [14] reported that a 4-week telerehabilitation regimen achieved comparable improvements in ankle function to traditional clinic-based therapy for patients with CAI. In acute ankle sprains, Figueroa-García et al [44,45] found that adding a home-based telerehab program to standard care significantly improved FAAM scores, and this approach was also shown to be cost-saving. These studies demonstrate that remote physiotherapy can yield outcomes comparable to or better than conventional rehabilitation while potentially lowering costs, aligning with this trial’s findings. Our study extends these observations by being the first to establish both noninferior clinical efficacy and greater cost-effectiveness of a digital rehabilitation intervention for CAI after surgery.

Patient adherence to the rehabilitation program was high in both groups, with the number of sessions performed per week slightly higher in the DT group (mean 3.8, SD 1.2) compared to the physiotherapy group (mean 3.6, SD 1.3), which was statistically significant (*P*<.001; see Table 6). This suggests that the in-home DT system did not negatively affect patient engagement. In terms of satisfaction, patients in both groups reported high levels of agreement with the intervention, exercise program adherence, pain relief, and functional improvement, with no significant differences between groups. This high level of satisfaction is crucial, as it indicates that patients were comfortable with the DT approach and felt it was effective [41]. Additionally, the ability to receive guidance and support remotely can empower patients to take an active role in their recovery, potentially leading to better long-term outcomes.

The North American Expert Consensus Group on best practices for acute rehabilitation after total hip replacement recommended in 2014 that trained health professionals (physiotherapists) provide supervised rehabilitation interventions shortly after discharge from an acute care facility [46]. While acknowledging global differences in rehabilitation practices and program delivery, over 75% of the panelists recommended individual therapy instead of group therapy in an outpatient setting or at home [46,47]. In Canada, Australia, and the United States, at least one-third of patients receive face-to-face home-care rehabilitation services, but in China, the shortage of trained physiotherapists means that only around 5% of postoperative total hip arthroplasty and total knee arthroplasty patients receive in-hospital or home-care rehabilitation services [46-49]. To address this issue, the DT system has emerged as a promising new method of service delivery in China.

The recovery of the DT group may have been influenced by several factors. The DT system focuses on educating patients about self-applied mobilization techniques and prioritizes exercise, providing patients with more opportunities to self-treat outside of formal physiotherapy sessions. This emphasis on education may have contributed to the higher technical proficiency in the home exercise program and improved patient compliance by targeting internal control points. As China’s younger population continues to develop an affinity for sports and engage in various physical activities, it is expected that the acceptance of the DT system will increase in future generations.

Given the increasing focus on value-based health care delivery by clinicians and service providers, it is imperative that future research efforts prioritize the development and assessment of DT system models that build upon well-established physiotherapy interventions. To facilitate the growth of DT systems, it is crucial to gain a deeper understanding of its potential for diverse demographic groups, including the elderly, those from different ethnic and educational backgrounds. Moreover, ensuring that new DT system models are readily transferable will increase adoption rates and lead to better patient care and choice.

Methodological factors such as the use of randomization, validated outcome measures, and low drop-out rates strengthen this study. However, certain limitations must be considered. Because the long-term effects of this rehabilitation program are unknown, the limited follow-up period of 24 weeks has implications for the interpretation of the results. Therefore, future studies must take advantage of the extended follow-up period to better characterize the long-term effects of this alternative service delivery model. Finally, the quality of the internet connection between DT system units is easily monitored in this controlled environment, a factor that can be variable when delivered to a patient’s home. For these reasons, this study should be viewed as a proof-of-principle study, and future studies should be conducted in communities and homes where patients are isolated to explore the impact of these factors.

Another possible limitation of the study was that the same experienced orthopedic physiotherapist conducted all the DT system sessions, while the control group was managed by multiple physiotherapists. Participants in the control group may not have received consistent care from the same physiotherapist throughout the study. This inconsistency in the therapist-patient relationship could have influenced participant satisfaction, as the intervention group may have developed a stronger bond with their therapist due to their consistency.

### Conclusion

In conclusion, for patients with CAI following modified Brostrom surgery, an individually tailored digital PAST program achieved functional outcomes comparable to conventional face-to-face physiotherapy, confirming its clinical noninferiority. The digital intervention also reduced rehabilitation costs per patient, demonstrating superior cost-effectiveness.

## Supplementary material

10.2196/78307 Multimedia Appendix 1 Protocol.

10.2196/78307 Multimedia Appendix 2 The physiotherapeutic ankle-specific training (PAST) program and staged goals following modified Brostrom surgery for chronic ankle instability.

10.2196/78307 Multimedia Appendix 3 Timeline of data collection.

10.2196/78307 Multimedia Appendix 4 Effectiveness estimates from linear mixed effects models, per protocol analysis.

10.2196/78307 Multimedia Appendix 5 Changes in outcomes for the digital training and physiotherapy groups at weeks 12 and 24 follow-up, per protocol analysis.

10.2196/78307 Checklist 1 CONSORT-EHEALTH checklist.

## Abbreviations

CAI: chronic ankle instability
CONSORT: Consolidated Standards of Reporting Trials
DT: digital training
FAAM: Foot and Ankle Ability Measure
FAAM-ADL: Foot and Ankle Ability Measure—Activities of Daily Living
FAAM-S: Foot and Ankle Ability Measure—Sports
ICC: intraclass correlation coefficient
ICER: incremental cost-effectiveness ratio
MBS: modified Brostrom surgery
MCID: minimal clinically important difference
PAST: physiotherapeutic ankle-specific training
