# Supplementary material for: Digital Physiotherapeutic Ankle-Specific Training System for Patients With Chronic Ankle Instability Following Modified Brostrom Surgery: Noninferiority Randomized Controlled Trial at a Tertiary Grade A Trauma Center in China
Source: JMIR Mhealth Uhealth. 2025 Dec 18;13:e78307. doi: 10.2196/78307 (PMC12741553; doi:10.2196/78307)
Supplement: Multimedia Appendix 1 [file mhealth-v13-e78307-s001.doc]

**Digital Physiotherapeutic Ankle-Specific Training System for Patients with Chronic Ankle Instability Following Modified Brostrom Surgery: A Noninferiority RCT at a Tertiary Grade A Trauma Center in China**

**Trial registration**: This clinical trial was registered at chichr.org.cn (ID: ChiCTR2300075292).

**Protocol version**: Version 1.0

**Principal investigator**: Xin Ma

**Research participants**: Xiang Gen, Yilin Jin, Yunxia Li, Xu Wang, Xin Zhang, Yiming Lv, Shengdi Lu

**Institute of Primary investigator**: Department of Orthopedics, Huashan Hospital, Shanghai, China

**Name and contact information for the trial sponsor**: Xin Ma.

**Role of sponsor**: This is an investigator initiated clinical trial. Therefore, the funder played no role in the design of the study; collection, analysis and interpretation of data; and in the decision to write and submit the manuscript for publication.

**Abstract**

**Background:** Functional rehabilitation is often employed for patients with chronic ankle instability (CAI) and digital training system is gaining popularity in postoperative functional rehabilitation, whether it can be successfully carried out in CAI remains uncertain. The purpose of this trail was to determine whether a home-based digital training system method is not clinically inferior to standard face-to-face rehabilitation for patients with CAI following modified Brostrom Surgery in China.

**Methods**: This study presents a single-blinded protocol for a non-inferiority randomized controlled trial. Eight participants following modified Brostrom surgery for CAI will be randomly divided into two groups: digital training system (TELE) and face-to-face (PT) circuit training with a 1:1 ratio. A standardized training protocol was implemented, consisting of three key components: stretching exercises, range of motion (ROM) exercises, and strength training. For patients in TELE group, the rehabilitation program was delivered through a smartphone APP (device: Joymotiontm software, Shanghai Medmotion Medical Management Co., Ltd., Shanghai, China.) which provided participants exercise instructions, feedback on their training performance, and the real-time two-way video and audio interaction with the PT. In PT group, patients visited designated PT clinics in Shanghai. Each patient in PT group visited 3 to 4 times each week to engaged to the exercise program for 12 weeks. The primary outcomes were symptoms and pain, collected at week 12. They were also collected in week 24 as secondary outcomes. Pain and symptom were measured by the Foot and Ankle Ability Measure (FAAM). Secondary outcomes include changes in other clinical outcomes, in morphological characteristics, adherence, acceptability, and treatment perspective.

**Discussion:** Standardized training through digital training system may contribute to developing effectiveness of rehabilitation of patients with CAI following modified Brostrom Surgery.

**Introduction**

Sports participation often leads to ankle sprains, a common injury^1,2^. However, sports enthusiast and athletes with lateral ankle sprains have a high reinjury rate of 70% to 80%, which can result in chronic ankle instability (CAI)^3,4^. After lateral ankle sprains, clinicians need to address various factors, such as muscle weakness, postural-control deficits, decreased range of motion, and frequent reinjury. The ultimate aim is to return patients to high levels of function despite a limited time frame^4-6^. Thus, clinicians face the challenge of finding effective approaches to improve ankle stability and prevent reinjury in order to treat Chronic Ankle Instability (CAI).

CAI is characterized by residual lateral instability, resulting in repeated ankle sprains^7^. This condition can be caused by functional ankle instability, mechanical ankle instability, or a combination of both^7^. Deficits in postural control and strength are also commonly observed after injury, along with structural laxity of the joint^8,9^. Recent studies have shown that aspects of both functional and mechanical ankle instability differ significantly in those with CAI when compared to matched healthy ankles^10^. Surgical intervention, such as the modified Brostrom surgery (MBS) is a well-accepted method, to restore mechanical stability^11^. On the other hand, effective rehabilitation post-surgery is now shifting towards a more functional approach, emphasizing dynamic, closed kinetic-chain activity instead of quiet standing and open-kinetic-chain positioning. However, specialized physical therapist (PT) cannot afford to this patient group in China. Since the growing rehabilitation needs of the adolescent after CAI cannot be met by the current labor force of PT in China, the exploration for new effective alternatives to ensure reliable and accessible postoperative physical rehabilitation is vital and urgent^12^.

One alternative approach to treatment is utilizing digital training system technology to administer rehabilitation programs to patients in their own homes. This method may offer a solution to the challenges of accessibility faced by patients residing in remote or rural regions, as well as those in urban areas with limited transportation options^13,14^. Following their discharge from the hospital, numerous patients who have undergone CAI encounter difficulties accessing healthcare services. Patients residing in rural regions encounter additional barriers to access due to the lengthy travel times and distances involved for both patients and healthcare providers. The adoption of technology-enabled home exercise programs (HEPs) could incentivize patients to engage in exercise routines more frequently, potentially rectifying the strength impairments often observed in CAI. In addition to resolving accessibility challenges, implementing such HEPs could also lead to cost savings in the provision of healthcare services.

Several studies have explored the use of digital training system in postoperative rehabilitation programs for total knee arthroplasty (TKA) and total hip arthroplasty (THA), demonstrating promising outcomes^15^. Not only have these programs been found to produce comparable results to traditional in-person rehabilitation, but the majority of patients enrolled in digital training system programs have expressed satisfaction with this alternative approach^15^. The goal of this study is to determine whether digital training system has similar clinical outcomes and cost-effectiveness as conventional face-to-face rehabilitation program for patients with CAI following MBS.

**Objective**

The primary objective of this randomized controlled trial is to evaluate whether rehabilitation exercises delivered through digital training system are as effective as those administered in person in alleviating pain and enhancing physical function at 12 weeks (primary endpoint) and 24 weeks (secondary endpoint) in individuals following modified Brostrom Surgery for CAI. The secondary objectives include assessing clinical outcomes such as muscle strength and other functional outcomes. Additionally, the study will examine the effectiveness, adherence, acceptability, and perceptions of treatment at both the 12-week and 24-week marks post-treatment. Safety will be monitored by documenting any adverse events throughout the intervention period.

**Trial design**

The present protocol is a non-inferiority randomized, parallel, single-blind trial with 1:1 allocation to the involved groups studied.

Participants in the experimental group will be assigned a 12-week training regimen via digital training system. Outcome assessments occurred at the onset (E0), 12 weeks (E1), and 24 weeks (E2). Stratified randomization will be employed to assign participants to one of two intervention groups, ensuring balanced distribution based on initial characteristics such as sex, age, education level, and insurance type. The randomization process will be overseen by a single investigator who had access to these characteristics but did not participate in recruitment or initial assessments. While participants and therapists will be aware of group assignments, the physiotherapists and investigators will be responsible for evaluating outcomes at each stage remained unaware of the allocations.

All patients will be provided written informed consent in compliance with the declaration of Helsinki prior to joining the study. All study protocols were approved by the Research Ethics Committee of Huashan Hospital (IRB no.:2022-069). This clinical trial was registered at chichr.org.cn (ID: ChiCTR2300075292).

**Methods: participants, interventions, and outcomes**

*Eligibility criteria*

Primary inclusion criteria:

(1) be awaiting MBS following a diagnosis of CAI, (2) be discharged from the hospital and returning home, (3) reside in an area with access to high-speed Internet services (with a minimum upload speed of 512 kb/s), and (4) live within a one-hour driving distance from the treating hospital.

Primary exclusion criteria:

(1) having health conditions that could interfere with the tests or rehabilitation program, including undergoing other lower-limb surgeries within the past nine months, (2) planning a second lower-limb surgery within the next four months, (3) experiencing cognitive or collaboration problems, and (4) encountering major postoperative complications.

Definitive exclusion criteria:

Participants will be excluded during the face-to-face evaluation if they:

Have cognitive impairment that compromise the understanding of the tests (obtained by Mini Mental State Examination (MMSE))^16^

Screening definitive eligible criteria:

Mini Mental State Examination (MMSE)

The Mini-Mental State Examination (MMSE) will serve as the screening tool for cognitive impairment, and it is a criterion for exclusion in this study. The MMSE is comprised of 11 items organized into two parts: the first part demands verbal responses and assesses guidance, memory, and attention; the second involves tasks related to reading and writing. The assessment is conducted sequentially as listed, with the potential to achieve a maximum score of 30. The following criteria will be used to identify cognitive impairment: individuals who are illiterate must score below 20, those with 1 to 4 years of education must score below 25, individuals with 5 to 8 years of education less than 26.5, those with 9 to 11 years of education less than 28, and individuals with more than 11 years of education less than 29^16^.

Intervention

A standard postoperative CAI approach was used. The standardized rehabilitation intervention was based on the recommendations of a group of experts^17-19^, including 3-4 sessions per week of forty-five minutes to sixty minutes; the intensity and duration were an assessment of the supervising PT according to each patient’s tolerance and needs (find details in supplemental materials). Advice concerning pain control, walking aids, and the return to activities was also given to the patients. The difficulty and intensity of the exercise were increased according to each patient’s tolerance and needs.

*Modes of Service Delivery*

In-Home Digital training system (TELE Group)

The rehabilitation program was delivered through a smartphone APP (Joymotion software, Shanghai Medmotion Medical Management Co., Ltd., Shanghai, China.) which provided participants exercise instructions, feedback on their training performance, and the real-time two-way video and audio interaction with the PT. The APP was installed by a technician on the same day of the patient’s discharge. Internet connection was provided by the patients’ own home Wi-Fi. PT at the rehabilitation department of Huashan hospital initiated the conference at the appointed time scheduled with the patient every week. The APP provides daily rehabilitation exercises with detailed instructions and records the exercise completion rates. The rehabilitation program was prescribed by the supervising PT and was assigned to the patient as “daily tasks”.

In-clinic physical therapist care (PT group)

Patients in the PT group visited the Medmotion Clinic 3 to 4 times each week for the 12 weeks after discharge, patients received a standard rehabilitation program by the physiotherapist. The components of the intervention and following home exercises were prescribed according to PT’s assessment before and after exercise.

During the intervention, participants received an explanation about the exercises and use of the APP and filled out questionnaires on the day of discharge. The second and third follow-ups were conducted at 12 weeks and 24 weeks, including physical tests and filling out questionnaires on the APP or at the clinic.

In-clinic PT care (PT group)

Patients in the PT group visited designated PT clinics in Shanghai. Each patient in PT group visited 3 to 4 times each week to engaged to the exercise program for 12 weeks. The components of the intervention and following home exercises were prescribed according to PT’s assessment before and after exercise.

Criteria for discontinuing or modifying allocated interventions

Participants may exit the study at any point and for any reason without any repercussions. Data collected prior to their withdrawal will still be included in the study's analysis.

Strategies to improve adherence to interventions

Adherence reminders will be issued during telephone calls to the Digital training system (TR) group and throughout in-person sessions for the Face-to-Face (PT) group. The researcher or physical therapist will enhance support by educating participants about the origins and symptoms of CAI, highlighting the advantages of physical activity. This educational effort will include strategies to encourage activity and manage pain during exercises, setting realistic expectations for possible results, reassuring participants that pain should not be a deterrent, and bolstering confidence in their ability to engage in physical activities effectively.

Provisions for post-trial care

Trial center insurance will compensate for those who suffer harm from trial participation.

**Outcomes**

*Primary outcomes*

The Foot and Ankle Ability Measure (FAAM) is a tool utilized for evaluating the overall self-reported functional status in patients with musculoskeletal injuries and disorders affecting the leg, ankle, and foot^20^. This measure comprises two subscales, namely Activities of Daily Living (FAAM-ADL) and Sport (FAAM-S), both of which are scored on a 0% to 100% scale, where a higher score indicates a better functional status^22^. The FAAM is considered a reliable questionnaire for assessing the function of adult patients with Chronic Ankle Instability (CAI)^23^. The reported Minimal Detectable Change (MDC) scores for FAAM-ADL and FAAM-S are 3.9% to 4.8% and 7.6% to 7.9%, respectively^24^. Moreover, the Minimal Clinically Important Difference (MCID) scores for an adult population receiving treatment for musculoskeletal disorders in the leg, ankle, or foot are 8 and 9 points for FAAM-ADL and FAAM-S, respectively^22^.

*Secondary outcomes*

Static and Dynamic Balance

Time-in-Balance Test

Participants assumed a natural standing posture with their hands on their hips and eyes closed. They were directed to maintain their balance on the designated limb while the examiner timed the duration of the stance in seconds. Each attempt was limited to a maximum of 60 seconds, and any movement of the testing foot or contact with the contralateral foot resulted in termination of the trial. Prior to the test trials, one practice trial was allowed for the patient to become familiar with the procedure. The test was repeated three times, with 30 seconds of rest provided between each trial, and the duration of the longest trial was analyzed. This test has been shown to be reliable and sensitive to rehabilitation (intraclass correlation coefficient [ICC] = 0.99), and the methodology was consistent with previous reports^25,26^.

Foot-Lift Test

Patients were instructed to stand on one foot, with their hands on their hips, and their eyes closed while maintaining an erect stance. During the 30-second test, the number of foot lifts was recorded, with any part of the foot leaving the floor considered a foot lift. Touching the floor with the contralateral foot was deemed an error. Patients were instructed to avoid removing their hands from their hips, opening their eyes, and touching the stance limb with the contralateral foot, but such actions were not regarded as errors. Before the test trials, one practice trial was permitted for patient familiarization. The test was conducted three times, with a 30-second rest between each trial, and the average number of foot lifts from the three trials was used for analysis. This test has been shown to be valid and responsive to rehabilitation (ICC = 0.99), and the methodology is consistent with previous reports^25,26^.

Star Excursion Balance Test

During the Star Excursion Balance Test, patients stood on their test limb and extended their reach as far as possible in each of five directions while maintaining their balance. The five directions evaluated were anterior, anteromedial, medial, posteromedial, and posterolateral. Each reach was made over a cloth tape measure securely attached to the floor, with the distance measured by the investigator in centimeters and normalized to the patient's non-test limb length. Patients were given four practice trials in each direction with a 5-minute rest before the test sessions^26^. The test was conducted three times in each direction, with a 10-second rest between trials. The average of the three trials for each direction was used for analysis. This test has been demonstrated to be valid and responsive to rehabilitation (ICC range = 0.81-0.93), and the methodology is consistent with previous reports^27-29^.

Functional Performance

Ankle dorsiflexion range of motion

A goniometer was used for measuring the ankle-dorsiflexion range of motion. The stationary arm of the goniometer is aligned with the fibula, the axis is placed at the lateral malleolus, and the moving arm follows the fifth metatarsal. Goniometry has ICC values around 0.80 to 0.90, indicating good reliability^30^.

Side-Hop Test

During the lateral hop test, patients hopped laterally 30 cm on their involved limb for 10 repetitions as quickly as possible. The time taken to complete the test was recorded to the nearest 0.01 second using a handheld stopwatch (model AX725 Pro Memory; Accusplit, Pleasanton, CA). Patients were given a single practice trial for familiarization before the test trials. The test was conducted twice on the involved limb, with a 60-second rest provided between trials. The shortest trial was used for analysis. This test has been shown to be valid and responsive to rehabilitation (ICC = 0.99), and the methodology is consistent with previous reports^24.30^.

Figure-8 Hop Test

During the figure-8 hop test, patients hopped over a 5-meter distance on their test limb in a figure-8 pattern, and the time taken to complete the test was recorded to the nearest 0.01 second using a handheld stopwatch. Patients were given a single practice trial for familiarization before the test trials. The test was conducted twice on the involved limb, with a 60-second rest provided between trials. The shortest trial was used for analysis. This test has been shown to be valid and responsive to rehabilitation (ICC = 0.98), and the methodology is consistent with previous reports^25.31^.

*Cost measures*Costs assessed in this study included intervention costs, other healthcare expenses, costs for paid and informal home care, as well as expenses related to work absenteeism, presenteeism, and lost productivity in unpaid tasks.

Intervention costs were gathered from the Hospital Information System (HIS) of Huashan Hospital and the online payment system of Shanghai Medmotion Medical Management Company. Additional healthcare expenses encompassed costs for primary healthcare (e.g., general practitioner visits), secondary healthcare (e.g., non-initial hospital visits), and both prescribed and over-the-counter medications, all of which were obtained from HIS.

Paid home care expenses were evaluated by participants' reports on the number of hours of paid care received, priced through direct inquiries at the 12-week and 24-week follow-ups. Informal care costs were derived from the total hours of assistance provided by family, friends, and volunteers, as reported by patients during outpatient follow-up visits. These costs were calculated by multiplying the total hours by the average hourly income in Shanghai.

Absenteeism and presenteeism costs were estimated using the Productivity Cost Questionnaire^32^. Absenteeism costs were determined by counting the number of sick days and valuing them through the Friction Cost Approach (FCA; friction period = 24 weeks) with gender-specific price weights^20^. Presenteeism costs were assessed by having participants rate their performance level on days they worked despite health issues, on a scale from 0 (completely incapacitated) to 10 (fully functional). The cost of presenteeism was then calculated using the formula: Presenteeism days = ((10 - performance level) / 10) * number of days with health complaints and valued by gender-specific price weights^21^.

Finally, costs for unpaid productivity losses were estimated by having participants report hours lost in performing unpaid tasks (e.g., chores, volunteer work, educational activities), which were then valued using the average hourly income in Shanghai^33^.

*Adherence and acceptability*

Adherence was measured using several indicators: the number of calls received, the attendance rate of control group participants at sessions, and the experimental group participants' reported completion of sessions^33^. Additionally, both groups rated their agreement with statements concerning adherence and acceptability on a scale from 0 ('strongly disagree') to 10 ('strongly agree'). Participants also provided qualitative assessments of their perceptions of the exercise protocol's outcomes^34^.

**Sample size**

Sample size was calculated based on the data of primary outcomes (FAAM-ADL and FAAM-S), by means of the noninferiority power calculation described by Jones et al.^35^

The subscales of FAAM were calculated separately for the sample size. The MCID values determined by Paulsen et al.^36^ was used for noninferiority margin (8 and 9 points for FAAM-ADL and FAAM-S). The intervention will be accepted as equivalent if the difference between two groups is less than MCID. Calculations were based on 80% power and a type-I error of 5% (α=0.05). The values of FAAM-ADL yielded the largest sample size of 30 per group, thus we set a sample size of 40 per group based on a 25% of dropout rate.

**Recruitment**

Participants were enrolled from the surgical waiting lists of podiatrists in Huashan hospital.

**Allocation**

Sequence generation

A computer-generated randomization list was created using SAS Proc Plan in SAS/STAT 9.3 (SAS Institute, Cary, North Carolina). The statistician prepared this list and distributed it to each site's clinical coordinator in a series of sealed envelopes. Subsequently, the study coordinator conducted the randomization in the presence of the patient.

**Concealment mechanism**

A single investigator, privy only to participants' baseline characteristics like sex, age, BMI, and education level, will be tasked with pairing participants as outlined in the sequence generation section. Subsequently, this investigator will conduct simple randomization using a designated website to assign the intervention type each participant receives (TELE or PT). Following this, the investigator will communicate the assigned intervention to both the participants and the physical therapist responsible for administering the interventions.

**Blinding**

Throughout the study, all evaluators and investigators were blinded to group assignments. Decisions regarding data analysis were made while the investigators remained unaware of these assignments. However, due to the nature of the intervention, blinding of the subjects and clinicians was not feasible.

**Data collection and management**

Assessments will be conducted face-to-face at Huashan Hospital. On the initial day (before surgery), participants will complete a questionnaire about baseline information and physical examination on ankle function. This initial assessment, lasting around one hour, will be conducted by the same physical therapist/researcher.

The second assessment, scheduled 12 weeks after surgery, will involve participants filling out the FAAM-ADL and FAAM-S questionnaire on an e-paper form, followed by a physical examination of the Time-in-Balance test, Foot-Lift test, Star Excursion Balance test and other function examination (ROM of ankle-dorsiflexion, Side-Hop test, and Figure-8 Hop test) by a trained evaluator who is blinded to the intervention groups.

The last assessment, scheduled 24 weeks after surgery, will also involve participants filling out the FAAM-ADL and FAAM-S questionnaire on an e-paper form, followed by a physical examination of the Time-in-Balance test, Foot-Lift test, Star Excursion Balance test and other function examination (ROM of ankle-dorsiflexion, Side-Hop test, and Figure-8 Hop test) by a trained evaluator who is blinded to the intervention groups.

Further details regarding the data collection methods for these study outcomes are described in the “Outcomes” section. Participants will self-evaluate adherence, acceptability, and treatment perspectives at the 12-week and 24-week endpoints, with related questions included on the first assessment day post-intervention.

All assessments, at baseline, 6 weeks, and 12 weeks, will be consistently conducted by the same physical therapist/researcher. The physical therapist/researcher, will be blinded to group allocations. Furthermore, all randomized participants will be invited to attend all study timepoints, regardless of their adherence to the interventions.

**Plans to promote participant retention and complete follow-up**

During recruitment, participants will be thoroughly briefed on the study's design and requirements, emphasizing the significance of completing the follow-up assessments. Participants are permitted to withdraw from the study at any point and for any reason without consequence. Nonetheless, if feasible, participants will be encouraged to attend the final assessments scheduled at 12 weeks and 24 weeks.

**Data management**

Upon verification for completeness, data will be inputted into an electronically secured, password-protected database by a study collaborator who is blinded to the study details. Subsequently, a different blinded collaborator will verify the accuracy of data transfer and coding. In accordance with local ethics committee guidelines, all research data will be preserved for a period of 10 years following the conclusion of the study.

**Confidentiality**

The present study data will be documented and archived using an identification code for each participant. All personal information will be stored in a locked, password-accessible database. Paper forms and exams report containing personal information will be locked in a lockable metal cabinet. Physical and electronic personal documents will be safeguarded by the Trial Coordinator for 10 years as recommended by the local ethics committee; after that, they will be destroyed and deleted.

**Statistical methods**

Patient data were coded and securely stored using the hospital’s electronic data capture system, hosted on local servers. The primary analysis focused on intention-to-treat (ITT) population and per protocol population was used for sensitivity analysis, multiple imputation was employed to handle missing follow-up data. Statistical analyses were conducted using SPSS Statistics version 24.0 (IBM Corp, Chicago, IL) and R version 4.3.2 (R Foundation for Statistical Computing, Vienna, Austria). Baseline data were reported as mean ± SD with 95% confidence intervals (CIs) unless otherwise specified. Intergroup differences in baseline characteristics were evaluated with independent sample t-tests for continuous variables and Fisher’s exact test for categorical data.

Following treatment, continuous outcomes, including questionnaire scores and functional test results, were analyzed as changes from baseline (e.g., E3 - E1). These outcomes were compared between intervention groups at each time point using a linear mixed model for repeated measures (nlme, version 3.1–163, in R), with changes from baseline as the primary metric. The model accounted for the interaction between time and intervention, adjusted for age and sex as fixed effects, and included participants as random effects. Differences between the least squares means of the groups were estimated at each time point (emmeans, version 1.9.0, in R). The gain in the TELE group was evaluated as noninferior only if intergroup mean difference and its one-sided 95% confidence interval (CI) were less than 8 and 9 points for FAAM-ADL and FAAM-S points at E3. On the basis of the methodology of a noninferiority randomized trial, we tested the null hypothesis (H0) of a group difference against the alternative that the two treatments are equivalent (H1) according to our noninferiority margin of 8 and 9 points for FAAM-ADL and FAAM-S. Results for each time point were reported as mean value with standard deviation (SD), and differences between groups were expressed as Coefficient with two-sided 95% confidence intervals (CIs).

The incremental cost-effectiveness ratio (ICER) was used to assess the cost-utility of TELE compared with PT.

The ICER is the differential costs and outcomes between the tele-rehabilitation (TELE) and the control (PT). The numerator in the cost-effectiveness ratio is the monetary cost of the TELE intervention minus the monetary cost of PT. The annual costs of the projects were calculated by converting the 24-week costs, the period used for implementation. The denominator is the FAAM-ADL and FAAM-S utility gained by TELE minus the FAAM-ADL and FAAM-S utility gained by PT. Bootstrapping was used for a pair-wise comparison of the mean costs and effects between the TELE and PT groups. CIs for the mean differences in effects were obtained by bootstrapping (1000 replications). The bootstrapped cost and effect pairs were also graphically represented on a cost-effectiveness plane^37^.

**Methods in analysis to handle protocol non-adherence and any statistical methods to handle missing data**

The primary and secondary outcomes will be assessed using an intention-to-treat-analysis. Multiple imputation method will be used to impute missing data.

**Plans to give access to the full protocol, participant-level data, and statistical code**

The full protocol, participant-level data, and statistical code can be made available by the Trial Coordinator upon reasonable request.

**Oversight and monitoring**

Composition of the coordinating center and trial steering committee

The coordinating center is located at Huashan Hospital and comprises the Trial Coordinator, the principal investigator, 6 physical therapists, 1 computer scientist, and 1 data analysts. The study team meets weekly. There is no trial steering committee or public involvement group.

**Composition of the data monitoring committee, its role, and reporting structure**

A data monitoring committee was not considered as this study adopts a low-risk intervention, and an interim analysis is not planned in this study.

**Adverse event reporting and harms**

Participants encountering any adverse events are directed to inform their physical therapist immediately. All such events, whether reported by the participant or noted by the physical therapist during sessions, will be documented. In this randomized controlled trial, adverse events are identified as any issues that arise during the study due to the assessments, the exercise protocol, or the guidance provided by the physical therapist.

**Frequency and plans for auditing trial conduct**

The research team convenes weekly to review the trial's progress and address any emerging issues. Study documentation, including informed consents, inclusion and exclusion criteria, and source data, undergoes quarterly reviews by an independent researcher who is not associated with the current study. Should any documents be missing or inconsistencies discovered, the local ethics committee is promptly notified.

**Plans for communicating important protocol amendments to relevant parties**

All amendments to the protocol will be communicated and approved by Research Ethics Committee of Huashan Hospital.

**Dissemination plans**

The results of this RCT will be disclosed completely in international peer-reviewed journals. Both positive and negative results will be reported. In addition, participants will receive a report with all the results of the study.

**References**

1. Hootman JM, Dick R, Angel J. Epidemiology of collegiate injuries for 15 sports: summary and recommendations for injury prevention initiatives. J Athl Train. 2007;42(2):311–319.
2. Seeber PW, Staschiak VJ. Diagnosis and treatment of ankle pain with the use of arthroscopy. Clin Podiatr Med Surg. 2002;19(4):509–517.
3. McKay G, Goldie P, Payne W, Oakes B. Ankle injuries in basketball: injury rate and risk factors. Br J Sports Med. 2001;35(2):103–108.
4. Smith R, Reishchl S. Treatment of ankle sprains in young athletes. Am J Sports Med. 1986;14:465–471.
5. Willems T, Witvrouw E, Delbaere K, Mahieu N, De Bourdeaudhuij I, De Clercq D. Intrinsic risk factors for inversion ankle sprains in male subjects: a prospective study. Am J Sports Med. 2005;33(3):415–423.
6. Willems T, Witvrouw E, Delbaere K, Philippaerts R, De Bourdeaudhuij I, De Clercq D. Intrinsic risk factors for inversion ankle sprains in females–a prospective study. Scand J Med Sci Sports. 2005;15(5):336–345.
7. Hertel J. Functional anatomy, pathomechanics, and pathophysiology of lateral ankle instability. J Athl Train. 2002;37(4):364–375.
8. Hertel J. Functional instability following lateral ankle sprain. Sports Med. 2000;29:361–371.
9. Tropp H. Commentary: Functional ankle instability revisited. J Athl Train. 2002;37(4):512–515.
10. Hubbard T, Kramer L, Denegar C, Hertel J. Contributing factors to chronic ankle instability. Foot Ankle Int. 2007;28(3):343–354.
11. Lei T, Qian H, Lei P, Hu Y. Lateral augmentation reconstruction system versus modified Brostrom-Gould procedure: A meta-analysis of RCTs. Foot Ankle Surg. 2021;27(3):263-270.
12. China to promote rehabilitation and assistive products industry. 2016 Available from: <https://english.www.gov.cn/policies/latest_releases/2016/10/27/content_281475476503586.htm>.
13. Hoogland, J., A. Wijnen, T. Munsterman, C.L. Gerritsma, B. Dijkstra, W.P. Zijlstra, et al., Feasibility and Patient Experience of a Home-Based Rehabilitation Program Driven by a Tablet App and Mobility Monitoring for Patients After a Total Hip Arthroplasty. JMIR Mhealth Uhealth, 2019. 7(1): p. e10342.
14. Wijnen, A., J. Hoogland, T. Munsterman, C.L. Gerritsma, B. Dijkstra, W.P. Zijlstra, et al., Effectiveness of a Home-Based Rehabilitation Program After Total Hip Arthroplasty Driven by a Tablet App and Remote Coaching: Nonrandomized Controlled Trial Combining a Single-Arm Intervention Cohort With Historical Controls. JMIR Rehabil Assist Technol, 2020. 7(1): p. e14139.
15. Moffet, H., M. Tousignant, S. Nadeau, C. Mérette, P. Boissy, H. Corriveau, et al., In-Home Digital training system Compared with Face-to-Face Rehabilitation After Total Knee Arthroplasty: A Noninferiority Randomized Controlled Trial. J Bone Joint Surg Am, 2015. 97(14): p. 1129-41.
16. Zacaron K, Dias J, Abreu N, Dias R. Nível de atividade física, dor e edema e suas relações com a disfunção muscular do joelho de idosos com osteoartrite. Rev Bras Fisioter. 2006;10(3):279–84.
17. Cho BK, Kim YM, Kim DS, et al. Outcomes of the Modified Brostrom Procedure Using Suture Anchors for Chronic Lateral Ankle Instability—A Prospective, Randomized Comparison between Single and Double Suture Anchors. J Foot Ankle Surg. 2013;52(1):9-15.
18. Li X, Lin TJ, Busconi BD. Treatment of chronic lateral ankle instability: a modified Brostrom technique using three suture anchors. J Orthop Surg Res. 2009;4:1-6.
19. Shahrulazua A, Ariff Sukimin MS, Tengku Muzaffar TMS, et al. Early functional outcome of a modified Brostrom-Gould surgery using a suture anchor technique. Foot Ankle Int. 2011;32(2):153-157.
20. Li X, Lin TJ, Busconi BD. Treatment of chronic lateral ankle instability: a modified Brostrom technique using three suture anchors. J Orthop Surg Res. 2009;4:1-6.
21. Shahrulazua A, Ariff Sukimin MS, Tengku Muzaffar TMS, et al. Early functional outcome of a modified Brostrom-Gould surgery using a suture anchor technique. Foot Ankle Int. 2011;32(2):153-157.
22. Martin RL, Irrgang JJ, Burdett RG, Conti SF, Van Swearingen JM. Evidence of validity for the Foot and Ankle Ability Measure (FAAM). Foot Ankle Int. 2005;26(11):968–983.
23. Carcia CR, Martin RL, Drouin JM. Validity of the Foot and Ankle Ability Measure in athletes with chronic ankle instability. J Athl Train. 2008;43(2):179–183.
24. McKeon PO, Wikstrom EA. Sensory-targeted ankle rehabilitation strategies for chronic ankle instability. Med Sci Sports Exerc. 2016;48(5):776–784.
25. Cain MS, Garceau SW, Linens SW. Effects of a four week biomechanical ankle platform system on balance in high school athletes with chronic ankle instability.J Sport Rehabil. 201;26(1):1–7.
26. Linens SW, Ross SE, Arnold BL, Gayle R, Pidcoe P. Posturalstability tests that identify individuals with chronic ankle instability. J Athl Train. 2014;49(1):15–23.
27. Gribble PA, Hertel J, Plisky P. Using the Star Excursion Balance Test to assess dynamic postural-control deficits and outcomes in lower extremity injury: a literature and systematic review. J Athl Train. 2012;47(3):339–357.
28. Hertel J, Miller SJ, Denegar CR. Intratester and intertester reliability during the Star Excursion Balance Tests. J Sport Rehabil. 2000;9(2):104–116.
29. Hertel J, Braham R, Hale SA, Olmsted-Kramer LC. Simplifying the Star Excursion Balance Test: analysis of subjects with and without chronic ankle instability. J Orthop Sports Phys Ther. 2006;36(3):131–137.
30. Konor MM, Morton S, Eckerson JM, Grindstaff TL. Reliability of three measures of ankle dorsiflexion range of motion. Int J Sports Phys Ther. 2012;7(3):279-287.
31. Docherty CL, Arnold BL, Gansneder BM, Hurwitz S, Gieck J. Functional-performance deficits in volunteers with functional ankle instability. J Athl Train. 2005;40(1):30–34.
32. Bouwmans C, Krol M, Severens H, et al. The iMTA productivity cost questionnaire: a standardized instrument for measuring and Valuing health-related productivity losses. Value Health 2015;18:753–758.
33. Piaggio G, Elbourne DR, Altman DG, Pocock SJ, Evans SJ, Group C; CONSORT Group. Reporting of noninferiority and equivalence randomized trials: an extension of the CONSORT statement. JAMA. 2006 Mar 8;295(10):1152-1160.
34. Hinman RS, Lawford BJ, Campbell PK, Briggs AM, Gale J, Bills C, et al. Telephonedelivered exercise advice and behavior change support by physical therapists for people with knee osteoarthritis: protocol for the telecare randomized controlled trial. Phys Ther. 2017;97:524–536.
35. Jones, B., P. Jarvis, J.A. Lewis, and A.F. Ebbutt, Trials to assess equivalence: the importance of rigorous methods. Bmj, 1996. 313(7048): p. 36-9.
36. Paulsen, A., E.M. Roos, A.B. Pedersen, and S. Overgaard, Minimal clinically important improvement (MCII) and patient-acceptable symptom state (PASS) in total hip arthroplasty (THA) patients 1 year postoperatively. Acta Orthop. 2014. 85(1): p. 39-48.
37. Circular of the Shanghai Municipal Bureau of Human Resources and Social Security on the Average Wages of Employed Persons in Full-Caliber Urban Units in the City in 2022. available: https://rsj.sh.gov.cn/.
