# Supplementary material for: Digital Physiotherapeutic Ankle-Specific Training System for Patients With Chronic Ankle Instability Following Modified Brostrom Surgery: Noninferiority Randomized Controlled Trial at a Tertiary Grade A Trauma Center in China
Source: JMIR Mhealth Uhealth. 2025 Dec 18;13:e78307. doi: 10.2196/78307 (PMC12741553; doi:10.2196/78307)
Supplement: Multimedia Appendix 2 [file mhealth-v13-e78307-s002.doc]

**Materials and Methods**

**Intervention**

*The physiotherapeutic ankle-specific training (PAST) program following modified Brostrom surgery for chronic ankle instability.*

| **Periods** | **Evaluation** | **Rehabilitation Content** | **Homework** | **Remarks** |
| --- | --- | --- | --- | --- |
| **Day 2 postoperatively**  **(Immobilized by plaster)** | Refer to the Patient's information form postoperative condition, wound condition, pain index, swelling degree, toe mobility; | Medical history record; evaluation; education; assignment of homework; | Heel slides (ankle) Supine hip abduction Toe exercises Long-arc quad Prone hamstring training Hip extension training non-weight-bearing sit-to-stand transfer Non-weight-bearing crutch walking | Sessions of homework completed: The therapist evaluates flexibly based on the patient's signs and health condition |
| **Day 14 postoperatively, (Plaster removed in outpatients)** | Inquire about current pain and swelling; check for sensory abnormalities; observe wound healing; check toe mobility; gait check with crutches | Check homework, use of crutches; activation of the quadriceps on the affected side; toe mobility exercises; lower limb strength exercises, attempt partial weight-bearing training | Heel slides (ankle) Toe exercises Long-arc quad Prone hamstring training Thigh front stretch Standing hip abduction Standing hip extension Knee support plank Side-lying clam exercise Ankle active dorsiflexion (collateral ligament) Ankle active plantarflexion (collateral ligament) Forward weight shift with boot | Sessions of homework completed: The therapist evaluates flexibly based on the patient's signs and health condition |
| **Week 4 postoperatively** | Observe wound healing, inquire about current pain and swelling, check toe and ankle mobility, and evaluate functional gait with crutches | Review homework, use of crutches, foot mobility exercises, lower limb strength exercises, progression to full weight-bearing training | Scar tissue mobilization Heel slides (ankle) Knee support plank Toe exercises Side-lying clam exercise Long-arc quad Ankle active dorsiflexion (collateral ligament) Ankle active plantarflexion (collateral ligament) Double-leg mini squats (using walking boot or A60 brace, both referred to as "ankle brace" hereafter) Walking with crutches while protected by ankle brace | Sessions of homework completed: The therapist evaluates flexibly based on the patient's signs and health condition |
| **Week 6 postoperatively** | Observe scar healing, inquire about current pain and swelling, check toe and ankle mobility, assess lower limb muscle strength, and observe functional gait with crutches | Review homework, attempt walking without crutches (using ankle brace), foot mobility exercises (include inversion and eversion), lower limb strength and balance training | Scar tissue mobilization Glute bridge Resistance band dorsiflexion Ankle active eversion Ankle active inversion Seated calf raises Double-leg 1/4 squats Single-leg standing exercises (ankle brace) Gradually attempt walking without the ankle brace | Sessions of homework completed: The therapist evaluates flexibly based on the patient's signs and health condition |
| **Week 8 postoperatively** | Inquire about current steps and pain, check foot mobility, assess lower limb muscle strength, and observe gait | Review homework, attempt walking without crutches and brace, calf muscle group-specific strength training, ankle mobility exercises, lower limb strength exercises, balance training | Single-leg glute bridge Ankle alphabet drawing Double-leg calf raises Good morning exercise Standing calf muscle stretch 1/4 squats with weight shift Resistance band 1/4 squat clamshell (0-45 degrees) Single-leg standing exercises Walking without crutches and ankle brace | Sessions of homework completed; The therapist evaluates flexibly based on the patient's signs and health condition |
| **Week 10 postoperatively** | Inquire about current steps and pain, check ankle mobility, assess lower limb muscle strength, and observe gait | Review homework, ankle mobility exercises, calf muscle group-specific exercises, lower limb strength exercises, balance training | Kneeling foot dorsum stretch Single-leg glute bridge Single-leg calf raises Single-leg deadlift Standing calf muscle stretch Lunge squats Ankle resistance crab walk Single-leg standing exercises | Sessions of homework completed: The therapist evaluates flexibly based on the patient's signs and health condition |

*Staged goals for PAST modified Brostrom surgery for chronic ankle instability*

| **Periods** | **Patient Weight Bearing** | **Goal of active ROM** | **Goal of function** |
| --- | --- | --- | --- |
| **Day 2 postoperatively**  **(Immobilized by plaster)** | Non-weight-bearing on the affected side | Maintenance of mobility in toes, hips, and knees | Non-weight-bearing crutch walking |
| **Day 14 postoperatively, (Plaster removed in outpatients)** | Partial weight-bearing on the affected side protected by ankle brace, full weight-bearing if no pain | Maintenance of mobility in toes, hips, and knees, start plantarflexion and dorsiflexion after plaster removal | Non-weight-bearing stair climbing |
| **Week 4 postoperatively** | Full weight-bearing protected by ankle brace | Start plantarflexion and dorsiflexion after plaster removal | Full weight-bearing walking with crutches protected by ankle brace |
| **Week 6 postoperatively** | Full weight-bearing with boots, gradually attempt walking without ankle brace (therapists may strengthen education during this stage) | Start inversion and eversion of the ankle | Walking without crutches with ankle brace or completely independent walking |
| **Week 8 postoperatively** | Full weight-bearing | Restore to 80%-90% of the full range | Normal walking without crutches or ankle brace, stair climbing |
| **Week 10 postoperatively** | Full weight-bearing | Restore to the full range | Increased single-leg strength, preparation for running and jumping |

ROM: range of motion
