# Supplementary material for: Digital Physiotherapeutic Ankle-Specific Training System for Patients With Chronic Ankle Instability Following Modified Brostrom Surgery: Noninferiority Randomized Controlled Trial at a Tertiary Grade A Trauma Center in China
Source: JMIR Mhealth Uhealth. 2025 Dec 18;13:e78307. doi: 10.2196/78307 (PMC12741553; doi:10.2196/78307)
Supplement: Multimedia Appendix 3 [file mhealth-v13-e78307-s003.docx]

**Table S1. Timeline of data collection.**

| **Time Point*** | **FAAM-ADL** | **FAAM-S** | **Time-in-Balance Test** | **Foot-Lift Test** | **Star Excursion Balance Test** | **Ankle dorsiflexion range of motion** | **Side-Hop Test** | **Figure-8 Hop Test** | **Cost Data Collected #** | **Adverse Events / Serious Adverse Events (continuous)** |
| --- | --- | --- | --- | --- | --- | --- | --- | --- | --- | --- |
| **Baseline (pre-surgery)** | ✓ | ✓ | ✓ | ✓ | ✓ | ✓ | ✓ | ✓ | - | - |
| **12 weeks (end of intervention)** | ✓ | ✓ | ✓ | ✓ | ✓ | ✓ | ✓ | ✓ | ✓ | ✓ |
| **24 weeks (final follow-up)** | ✓ | ✓ | ✓ | ✓ | ✓ | ✓ | ✓ | ✓ | ✓ | ✓ |

FAAM-ADL= The Foot and Ankle Ability Measure-activities of daily living; FAAM-S= The Foot and Ankle Ability Measure-sport.

*The table below outlines when each outcome measure was assessed in the trial. Participants were evaluated at baseline (pre-surgery), 12 weeks postoperatively (end of the intervention), and 24 weeks postoperatively (final follow-up). A check mark (✓) indicates that the corresponding variable was collected at that time point. Note that cost data (combining all cost domains) were gathered only at the 12-week and 24-week follow-ups, and adverse events were monitored continuously throughout the study (recorded during the follow-up visits).

#Costs assessed in this study included intervention costs, other healthcare expenses, costs for paid and informal home care, as well as expenses related to work absenteeism, presenteeism, and lost productivity in unpaid tasks.

Intervention costs were gathered from the Hospital Information System (HIS) of Huashan Hospital and the online payment system of Shanghai Medmotion Medical Management Company. Additional healthcare expenses encompassed costs for primary healthcare (e.g., general practitioner visits), secondary healthcare (e.g., non-initial hospital visits), and both prescribed and over-the-counter medications, all of which were obtained from HIS.

Paid home care expenses were evaluated by participants' reports on the number of hours of paid care received, priced through direct inquiries at the 12-week and 24-week follow-ups. Informal care costs were derived from the total hours of assistance provided by family, friends, and volunteers, as reported by patients during outpatient follow-up visits. These costs were calculated by multiplying the total hours by the average hourly income in Shanghai.
