# Supplementary material for: Digital Physiotherapeutic Ankle-Specific Training System for Patients With Chronic Ankle Instability Following Modified Brostrom Surgery: Noninferiority Randomized Controlled Trial at a Tertiary Grade A Trauma Center in China
Source: JMIR Mhealth Uhealth. 2025 Dec 18;13:e78307. doi: 10.2196/78307 (PMC12741553; doi:10.2196/78307)
Supplement: Multimedia Appendix 4 [file mhealth-v13-e78307-s004.doc]

**Table S1. Effectiveness estimates from linear mixed effects models, per protocol analysis.**

| **Outcome** | **12 weeks follow-up** | | | **24 weeks follow-up** | | |
| --- | --- | --- | --- | --- | --- | --- |
|  | **Coefficient** | **95% CI** | **P value** | **Coefficient** | **95% CI** | **P value** |
| **PROMS** |  |  |  |  |  |  |
| FAAM-ADL | 0.308 | (-1.04, 1.66) | 0.655 | 0.051 | (-1.30, 1.40) | 0.941 |
| FAAM-S | -0.641 | (-2.75, 1.47) | 0.551 | 0.590 | (-1.52, 2.70) | 0.584 |
| **Time-in-Balance Test (s)** | -1.885 | (-2.66, -1.11) | <0.001 | -0.869 | (-1.65, -0.09) | 0.029 |
| **Foot-Lift Test (times)** | -0.897 | (-1.28, -0.51) | <0.001 | -0.923 | (-1.31, -0.54) | <0.001 |
| **Star Excursion Balance Test** |  |  |  |  |  |  |
| Anterior (cm) | -1.026 | (-1.50, -0.55) | <0.001 | -1.264 | (-1.74, -0.79) | <0.001 |
| Posteromedial (cm) | -0.149 | (-1.06, 0.76) | 0.749 | 0.108 | (-0.80, 1.02) | 0.817 |
| Posterolateral (cm) | -1.672 | (-2.74, -0.60) | 0.002 | -2.087 | (-3.16, -1.02) | <0.001 |
| **Function** |  |  |  |  |  |  |
| Ankle-dorsiflexion range of motion (°) | -0.256 | (-0.44, -0.07) | 0.007 | 0.026 | (-0.16, 0.21) | 0.789 |
| Side-Hop Test (s) | -0.195 | (-0.51, 0.12) | 0.228 | -0.251 | (-0.57, 0.07) | 0.120 |
| Figure-8 Hop Test (s) | 0.787 | (0.61, 0.97) | <0.001 | 0.951 | (0.77, 1.13) | <0.001 |

DT = Individually tailored physiotherapeutic ankle-specific training (PAST) via a digital training system; PT = Conventional physiotherapist face-to-face training; PROM=Patients reported outcome measures; FAAM-ADL= The Foot and Ankle Ability Measure-activities of daily living; FAAM-S= The Foot and Ankle Ability Measure-sport
