# Supplementary material for: Digital Physiotherapeutic Ankle-Specific Training System for Patients With Chronic Ankle Instability Following Modified Brostrom Surgery: Noninferiority Randomized Controlled Trial at a Tertiary Grade A Trauma Center in China
Source: JMIR Mhealth Uhealth. 2025 Dec 18;13:e78307. doi: 10.2196/78307 (PMC12741553; doi:10.2196/78307)
Supplement: Multimedia Appendix 5 [file mhealth-v13-e78307-s005.doc]

**Table S1. Changes in outcomes for the DT and PT groups at weeks 12 and 24 follow-up, per protocol analysis.**

| **Outcome** | **12 weeks follow-up** | | | **24 weeks follow-up** | | |
| --- | --- | --- | --- | --- | --- | --- |
|  | **DT group (N=39)** | **PT group (N=39)** | **P value** | **DT group (N=39)** | **PT group (N=39)** | **P value** |
| **PROMS** |  |  |  |  |  |  |
| FAAM-ADL | 8.77 (3.44) | 8.46 (3.25) | 0.686 | 9.33 (3.74) | 9.28 (3.95) | 0.953 |
| FAAM-S | 12.18 (3.42) | 12.82 (1.02) | 0.265 | 17.54 (6.56) | 16.95 (4.81) | 0.652 |
| **Time-in-Balance Test (s)** | 15.15 (1.84) | 17.04 (1.38) | <0.001 | 25.74 (1.79) | 26.61 (1.86) | 0.039 |
| **Foot-Lift Test (times)** | 3.03 (1.06) | 3.92 (0.81) | <0.001 | 3.74 (1.12) | 4.67 (0.93) | <0.001 |
| **Star Excursion Balance Test** |  |  |  |  |  |  |
| Anterior (cm) | 6.36 (1.02) | 7.38 (0.83) | <0.001 | 9.11 (1.42) | 10.37 (1.39) | <0.001 |
| Posteromedial (cm) | 10.83 (2.05) | 10.98 (2.53) | 0.776 | 13.47 (2.64) | 13.37 (2.56) | 0.855 |
| Posterolateral (cm) | 11.24 (2.86) | 12.91 (2.69) | 0.010 | 14.52 (3.11) | 16.61 (3.02) | 0.004 |
| **Function** |  |  |  |  |  |  |
| Ankle-dorsiflexion range of motion (°) | 2.28 (0.46) | 2.54 (0.51) | 0.021 | 3.28 (0.46) | 3.26 (0.44) | 0.802 |
| Side-Hop Test (s) | -4.33 (0.81) | -4.14 (0.71) | 0.262 | -5.57 (0.90) | -5.32 (0.97) | 0.239 |
| Figure-8 Hop Test (s) | -1.82 (0.44) | -2.61 (0.36) | <0.001 | -2.92 (0.55) | -3.87 (0.46) | <0.001 |

DT = Individually tailored physiotherapeutic ankle-specific training (PAST) via a digital training system; PT = Conventional physiotherapist face-to-face training; ITT = Intention-to-treat; PROM=Patients reported outcome measures; FAAM-ADL= The Foot and Ankle Ability Measure-activities of daily living; FAAM-S= The Foot and Ankle Ability Measure-sport
